# Changes in Epidemics of Respiratory Viral Infections Resulted From the COVID‐19 Pandemic in Shanghai

**DOI:** 10.1002/jmv.70034

**Published:** 2024-11-08

**Authors:** Chuchu Ye, Yao Tian, Dazhu Huo, Ting Zhang, Li Zhang, Bing Zhao, Yifeng Shen, Xinli Jiang, Xuancheng Hu, Haiyang Zhang, Lipeng Hao, Zhongjie Li, Li‐Qun Fang

**Affiliations:** ^1^ Shanghai Pudong New Area Center for Disease Control and Prevention Shanghai People's Republic of China; ^2^ State Key Laboratory of Pathogen and Biosecurity Academy of Military Medical Science Beijing People's Republic of China; ^3^ School of Health Policy and Management Chinese Academy of Medical Sciences (CAMS) & Peking Union Medical College Beijing People's Republic of China; ^4^ School of Population Medicine and Public Health Chinese Academy of Medical Sciences (CAMS) & Peking Union Medical College Beijing People's Republic of China; ^5^ Central Theater Command Center for Disease Control and Prevention Beijing People's Republic of China

**Keywords:** COVID‐19, respiratory viral infections, sentinel surveillance

## Abstract

To investigate the changing patterns of respiratory viral infections within the context of COVID‐19 pandemic. The etiological surveillance data of eight respiratory viral pathogens among patients with ARIs in Shanghai between 2013 and 2023 were analyzed to evaluate the dynamic patterns of respiratory viral infections in Shanghai compared to global other regions during pre‐pandemic (period 1), pandemic (period 2), and post‐pandemic (period 3) periods of COVID‐19. In Shanghai and various other global regions, there was a delay of 2‒4 months in the peak positive rate of IFV and a reverse seasonality for RSV, HMPV, and HBoV was observed following the relaxation of NPIs. The proportion of patients infected with any of these eight viruses experiencing fever or high fever notably increased. During the entire study period, IFV was consistently identified as the most prevalent virus, with IFV‐B as the predominant stain during period 2, and IFV‐A regained its dominance following the lifting of NPIs. The proportion of RSV among children significantly increased during period 2 compared to period 1. With the relaxation of NPIs, there has been a resurgence of certain viral pathogens, accompanied by notable alterations in seasonal patterns and the spectrum of viral pathogens.

## 1 Introduction

The COVID‐19 pandemic caused by SARS‐CoV‐2 has prompted global attention toward respiratory infections as a critical public health concern [1]. Governments worldwide have implemented various non‐pharmaceutical interventions (NPIs), such as social distancing, mask‐wearing, and lockdown measures, to mitigate the transmission of the virus [2]. The impact of these NPIs on epidemics caused by respiratory pathogens has been multifaceted. For instance, studies have demonstrated that the implementation of lockdowns and other public health measures has been associated with a decrease in the incidence of influenza‐like illness (ILI) and hospitalizations due to respiratory syncytial virus (RSV) in specific contexts [3, 4]. Furthermore, the reduction in social contact and exposure to respiratory pathogens may have resulted in alterations in the frequency and severity of non‐SARS‐CoV‐2 other respiratory infections [5]. In response to the evolving dynamics of the COVID‐19 pandemic, China officially lifted its “zero‐COVID” containment policy at the end of 2022 by implementing “the 20 measures” on November 1 and additional “10 measures” on December 7, 2022. The winter of 2023 in China was projected to witness a significant upsurge in respiratory infections, as it marked the first time without COVID‐19 restrictions since the onset of the pandemic in 2020 [6, 7]. The winter of 2023 observed a notable increase in hospitalizations attributed to prevalent respiratory infections during the cold season in China, which has garnered extensive attention. While similar phenomena have been documented in studies from other cities or countries, there is ongoing debate regarding the underlying causes for this occurrence [8, 9, 10].

The surveillance of acute respiratory infections (ARIs) in China is conducted through the National Influenza Surveillance Network. However, there is a lack of comprehensive multi‐pathogen‐related information [11]. To enhance the surveillance of respiratory pathogens, a nationwide ARI etiological surveillance program was established in 2009 and is officially maintained by the Chinese Center for Disease Control and Prevention (China CDC); however, this initiative was discontinued in early 2021 [12]. The ongoing etiological surveillance of ARIs in Shanghai City, located in eastern China, presents an opportunity to compare the epidemiology of etiological infections among ARI patients pre‐ and post‐COVID‐19 pandemic. This study aims to investigate the epidemic patterns of respiratory viruses subsequent to the relaxation of NPIs, utilizing etiological surveillance data on ARIs in Shanghai and comparing them with other global regions. Additionally, it aims to assess the impact of the COVID‐19 pandemic on the transmission dynamics of other respiratory viruses. Understanding these changes is crucial for informing public health policy and resource allocation in a post‐pandemic scenario.

## 2 Methods

### 2.1 Hospital‐Based Surveillance Network

From January 2013 to December 2023, an active etiological surveillance program for ARIs was implemented in 12 hospitals located in Shanghai City. These hospitals include Shanghai Children's Medical Center, Shanghai Sixth People's Hospital, Shanghai Pudong Hospital, Shanghai Gongli Hospital, Shanghai East Hospital, Shanghai Zhoupu Hospital, Shanghai Shuguang Hospital, Shanghai Renji Hospital (east), Shanghai Nicheng Community Health Service Center, Shanghai Yangshi Hospital, Shanghai Sixth people's Hospital (east), and Shanghai Zhangjiang Community Health Service Center. The standard operating protocol (SOP) for surveillance developed by China CDC is consistently implemented across all participating hospitals. It encompasses comprehensive guidelines for patient enrollment, specimen collection, laboratory testing, data recording, and management. The SOP remained unchanged throughout the surveillance period to maintain procedural consistency.

### 2.2 Patient Enrollment and Specimen Collection

The ARIs were defined as follows: (1) acute onset within 10 days; (2) at least one of the following symptoms/signs: sore throat, cough, expectoration, nasal congestion, runny nose, chest pain, tachypnoea, and abnormal pulmonary breath sounds; and (3) with or without fever. According to the pre‐established sample size specified by the SOP of surveillance, each hospital enrolled ARI patients from various departments, including internal medicine, emergency department, fever department, pneumology department, and infectious diseases department at a fixed time every week. For each week's enrollment in this study via convenience sampling method in sentinel hospitals, approximately 3‒10 patients with ARIs were included. In 2013 and 2023, respectively, due to the pilot phase of surveillance and the impact of the COVID‐19 pandemic on the data collection process resulted in smaller sample sizes. The details regarding the sample collection have been described in our previous publication [13]. To ensure the accuracy of respiratory specimens, nasopharyngeal swabs were obtained from patients before any therapeutic interventions. Testing was conducted within 24 h of collection; otherwise, samples were stored at −70°C until testing could be performed. For long‐distance transportation, all samples were transported using dry ice until they reached at the reference laboratory for subsequent testing.

### 2.3 Laboratory Testing Process

The nasopharyngeal specimens were utilized for the detection of eight viral pathogens, including influenza virus (IFV), RSV, human parainfluenza virus (HPIV), human metapneumovirus (HMPV), human coronavirus other than SARS‐CoV, MERS‐CoV, and SARS‐CoV‐2 (referred to as HCoV in this study), human rhinovirus (HRV), human adenovirus (HAdV), and human bocavirus (HBoV) using reverse transcriptase‐polymerase chain reaction (RT‐PCR) or PCR. Additionally, testing for SARS‐CoV‐2 was also conducted on patients enrolled between 2020 and 2023. According to the SOP, RT‐PCR was utilized for the detection of IFV, RSV, HPIV, HMPV, HCoV, HRV, and SARS‐CoV‐2, while PCR was used for detecting HAdV and HBoV. Subtyping of positive samples of IFV, RSV, HPIV, and HCoV was performed using real‐time RT‐PCR with specific primers and probes. Nucleic acid extraction could be performed using automated nucleic acid extraction equipment from Roche/Qiagen/bioMerieux/Applied BioSystem company or commercial kits from Invitrogen/Roche/Qiagen/Promega/Takara as well as other traditional methods. The primer/probes used and amplification conditions were consistent with our previous study [12]. All steps of nucleic acid extraction and PCR testing were carried out simultaneously with appropriate positive and negative controls.

### 2.4 Etiological Surveillance Data of ARIs in Other Cities or Countries

To compare the epidemic dynamics of respiratory viral infections in Shanghai City with those in other cities or countries in the context of the COVID‐19 pandemic, we also gathered etiological surveillance data on respiratory viral infections from Hong Kong and various global other locations across Europe, America, and Oceania. The Centre for Health Protection of Hong Kong provided data on six viral pathogens (IFV, RSV, HPIV, HMPV, HRV, and HAdV) detected among patients with ARIs, tested using molecular methods for etiological surveillance in Hong Kong between 2013 and 2023. However, due to the impact of the COVID‐19 pandemic, surveillance data exclusively focused on five pathogens (RSV, HPIV, HMPV, HRV, and HAdV) among patients under 18 years old from the 8th week of 2020 to the 29th week of 2022. Nevertheless, data regarding IFV detection was provided for ARI patients across all age groups. The Centre for Immunization and Respiratory Infectious Diseases of Canada provided the corresponding data from Ontario, Canada (2014−2023) (https://www.canada.ca/en/). The data of Ontario was collected by the Respiratory Virus Detection Surveillance System (RVDSS), a sentinel laboratory surveillance system comprising provincial, territorial, and regional public health laboratories as well as some hospital laboratories. These laboratories report both the total number of respiratory virus tests conducted and the corresponding number of positive results. The data of England, including RSV detection data starting from June 2019 and detection data for five other viral pathogens beginning in January 2017, was provided by the UK Health Security Agency (https://www.gov.uk/), which reported the percentage of individuals who tested positive among those with symptoms tested at sentinel laboratories through the Respiratory DataMart System. The data of IFV detection (2013−2023) from selected locations, including the United States, Australia, France, and Germany, were obtained from FluNet (www.who.int/tools/flunet), an influenza surveillance platform developed by WHO. FluNet provides publicly accessible information regarding laboratory‐confirmed influenza cases per participating country compiled on a weekly basis [14]. These data are contributed by all participating countries in the Global Influenza Surveillance and Response System (GISRS), as well as other national influenza reference laboratories collaborating with GISRS, and from WHO regional databases [15]. Data on RSV detection (2016−2023) from selected countries of the European Union and the European Economic Area (EU/EEA), including France, Germany, Ireland, and the Netherlands, were obtained from the European Respiratory Virus Surveillance Summary (ERVISS, www.erviss.org), established by the European Centre for Disease Prevention and Control (ECDC). The ARI pathogens data for these cities and countries encompassed weekly counts of detected viral pathogens and positive samples. However, for the UK only weekly positive rates of viral pathogens were provided. The entire process of data collection and processing has been illustrated in Supporting Information S1: Figure S1.

### 2.5 Data Management and Statistical Analysis

The demographic information, clinical manifestations, laboratory test results, and medication use data for patients with ARIs were collected through a comprehensive review of medical records and on‐site interviews. Trained clinicians then uploaded all the data to an online standardized database management system developed by the China CDC. Each case record was thoroughly examined to identify redundancies or incompleteness. Data collection was authorized by the National Health Commission of the People's Republic of China, and verbal informed consent was obtained from patients or their legal guardians. The surveillance protocol underwent a comprehensive review and received approval from the ethics review committee of the Shanghai Pudong CDC (Approval Number: 20230605‐001).

The study population was stratified into three age groups, including children (< 15 years old), adults (15–59 years old), and elderly (≥ 60 years old). The study period in Shanghai was divided into three distinct time periods to distinguish between different stages of the COVID‐19 pandemic: pre‐COVID‐19 pandemic (period 1, 2013 to 2019), during COVID‐19 pandemic (period 2, 2020 to 2022), and post‐COVID‐19 pandemic (period 3, starting from 2023). This categorization was based on the local progression of the COVID‐19 pandemic and the Oxford stringency index, which indicates the time when the stringent NPIs are lifted. Similarly, other global regions were also segmented into three separate study periods (Supporting Information S1: Figure S2). The clinical, etiological, and epidemiological features of patients with ARIs were compared across different study periods using Pearson's Chi‐square test or Fisher's exact test. The age‐specific patterns of IFV infection were examined using Join‐Point regressions (JPRs), as it has the highest prevalence among all viral infections. All statistical analyses were conducted in R version 4.2.1. with a significance level set at *p* < 0.05.

## 3 Results

### 3.1 Characteristics of Patients With ARIs in Shanghai

From January 1, 2013, to December 30, 2023, a total of 15 134 patients diagnosed with ARIs were enrolled in this study. The median age of these patients was 31 years (interquartile ranges, IQR: 8‒60 years), with 34.88% of them being under the age of 15 years and 33.98% requiring hospitalization. The proportion of male patients among all ARI cases was 54.82%. The top five symptoms reported among ARI patients throughout the study period were fever (78.68%), cough (72.96%), sore throat (38.63%), sputum (29.10%), and runny nose (24.99%) (Table 1). A significant increase in the proportion of male patients was observed during period 2, while a notable rise in the proportion of female patients was shown in period 3. Regarding age composition, there was a marked increase in the proportion of adults during period 2 accompanied by a decline in the proportion of elderly people (≥60 years old). Conversely, there was a substantial rise in the proportion of children (<15 years old) during period 3. Compared to period 1, an increase in the proportion of patients with fever (≥37.3°C) or high fever (≥39.1°C) was observed during period 3, while six symptoms including cough, runny nose, sputum, tachypnea, headache, and fatigue exhibited significant decreases (Table 1).

**Table 1:** Demographic characteristics and symptoms of patients with ARIs during the three periods associated with the COVID‐19 pandemic.

	Total		Period 1		Period 2		Period 3	
	No of cases	Proportion (%)	No of cases	Proportion (%)	No of cases	Proportion (%)	No of cases	Proportion (%)
Demographic characteristics	15 134		8882		3464		2788	
Sex								
Male	8297	54.82	4811	54.17	2020	58.31	1466	52.58
Female	6837	45.18	4071	45.83	1444	41.69	1322	47.42
Age, years								
0‒14	5279	34.88	2999	33.76	1221	35.25	1059	37.98
15‒59	6036	39.88	3491	39.30	1507	43.50	1038	37.23
≥ 60	3819	25.23	2392	26.93	736	21.25	691	24.78
Case type								
Inpatients	5142	33.98	3122	35.15	968	27.94	1052	37.73
Outpatients	9992	66.02	5760	64.85	2496	72.06	1736	62.27
Symptoms								
Fever (≥ 37.3°C)	11 454	78.68	6760	76.11	2585	74.62	2109	95.39
High fever (≥ 39.1°C)	1889	12.98	939	10.57	478	13.8	472	21.35
Cough	11 042	72.96	7191	80.96	1917	55.34	1934	69.37
Runny nose	3782	24.99	2577	29.01	669	19.31	536	19.23
Sore throat	5847	38.63	3501	39.42	1256	36.26	1090	39.10
Sputum	4404	29.10	2775	31.24	866	25.00	763	27.37
Chest pain	394	2.60	271	3.05	34	0.98	89	3.19
Tachypnea	926	6.12	727	8.19	83	2.40	116	4.16
Dyspnea	240	1.59	145	1.63	22	0.64	73	2.62
Headache	2248	14.85	1840	20.72	218	6.29	190	6.81
Fatigue	2547	16.83	1852	20.85	384	11.09	311	11.15
Abdominal pain	118	0.78	46	0.52	41	1.18	31	1.11
Diarrhea	152	1.00	55	0.62	71	2.05	26	0.93

### 3.2 Changing Seasonality of Viral Infections Resulted From COVID‐19 Pandemic or NPIs

**Figure 1:**
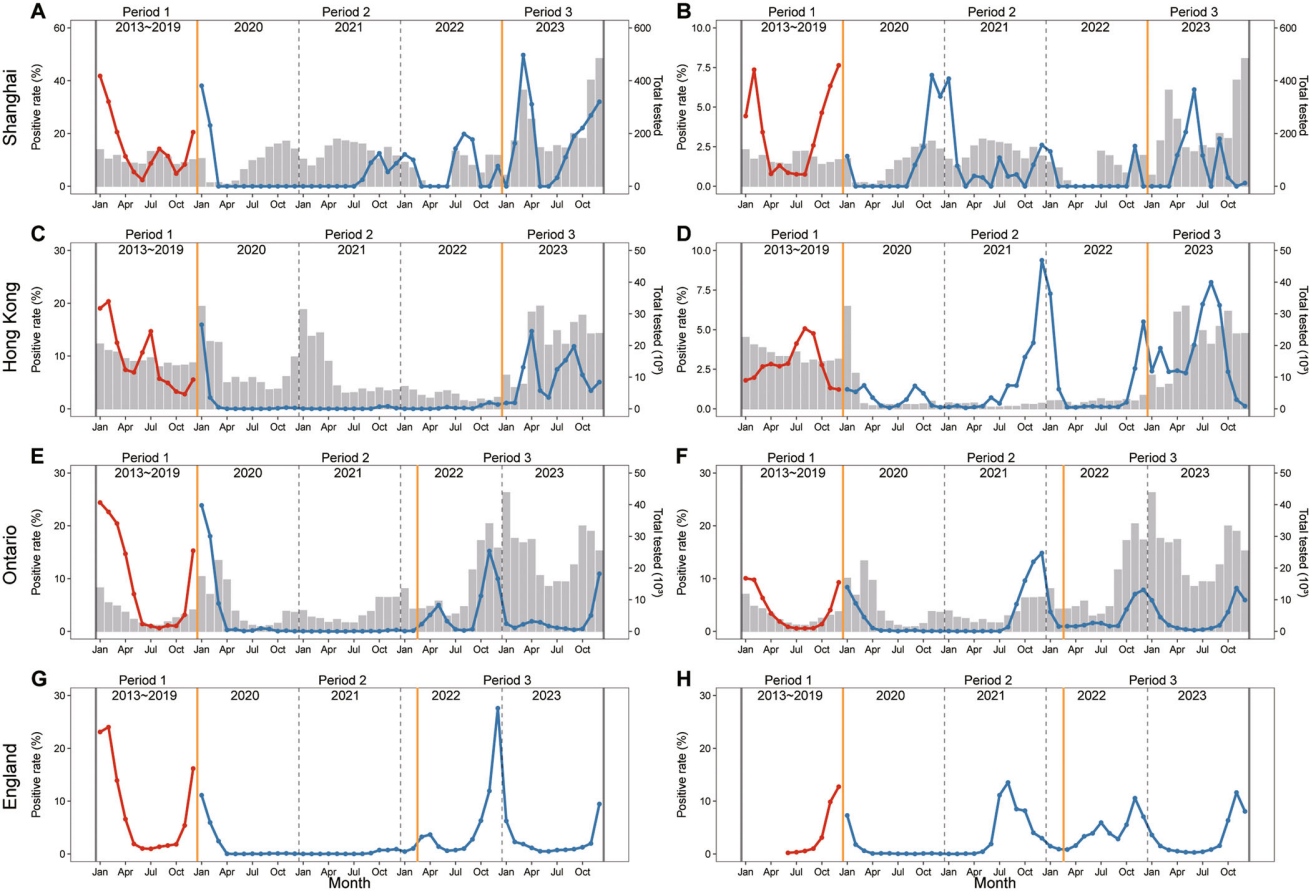
Seasonal patterns of IFV and RSV infection among patients with ARIs across different regions. (A) IFV in Shanghai. (B) RSV in Shanghai. (C) IFV in Hong Kong. (D) RSV in Hong Kong. (E) IFV in Ontario. (F) RSV in Ontario. (G) IFV in England. (H) RSV in England. The red line indicates the monthly average positive rate during period 1 (pre‐COVID‐19 pandemic, 2013−2019). The blue line represents the monthly positive rate from 2020 to 2023. The gray bar indicates the monthly total number of patients with AIRs tested. The orange solid line delineates the distinct periods for various regions. The dashed line delineates the different years. Due to the COVID‐19 pandemic, starting from the 8th week of 2020 through the 29th week of 2022, the official website of Hong Kong exclusively presented data on RSV detection among patients under 18 years old with ARIs, while including data on IFV detection among patients of all ages with ARIs.

**Figure 2:**
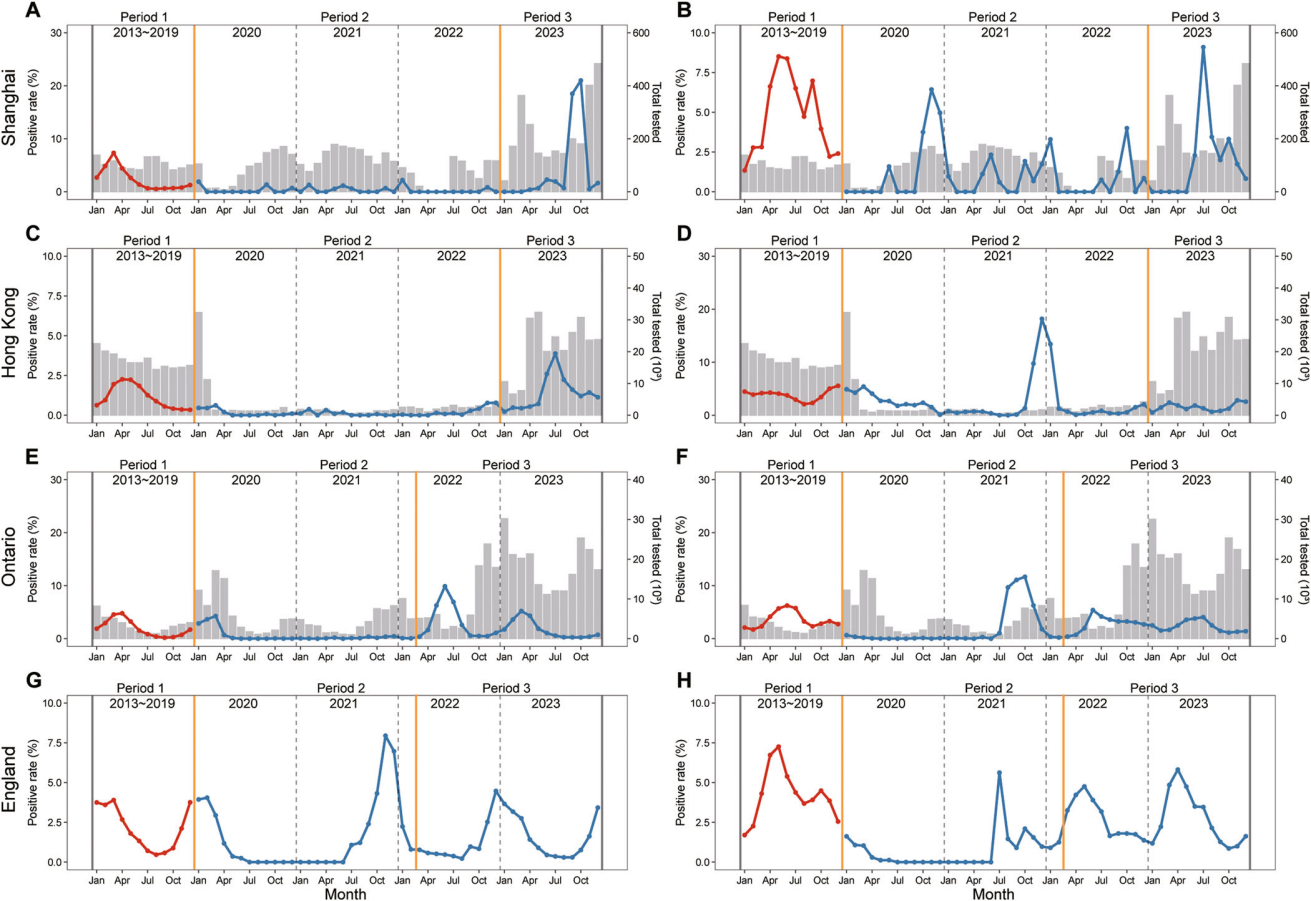
Seasonal pattern of HMPV and HPIV in different regions. (A) HMPV in Shanghai. (B) HPIV in Shanghai. (C) HMPV in Hong Kong. (D) HPIV in Hong Kong. (E) HMPV in Ontario. (F) HPIV in Ontario. (G) HMPV in England. (H) HPIV in England. The red line indicates the monthly average positive rate during period 1 (pre‐COVID‐19 pandemic, 2013−2019). The blue line represents the monthly positive rate from 2020 to 2023. The gray bar indicates the monthly total number of patients with AIRs tested. The orange solid line delineates the distinct periods for various regions. Due to the COVID‐19 pandemic, from the 8th week of 2020 through the 29th week of 2022, the website of Hong Kong only provided the data of HMPV and HPIV detection of ARIs patients under 18 years old. Due to the COVID‐19 pandemic, starting from the 8th week of 2020 through the 29th week of 2022, the official website of Hong Kong exclusively presented data on HMPV and HPIV detection among patients under 18 years old with ARIs.

**Figure 3:**
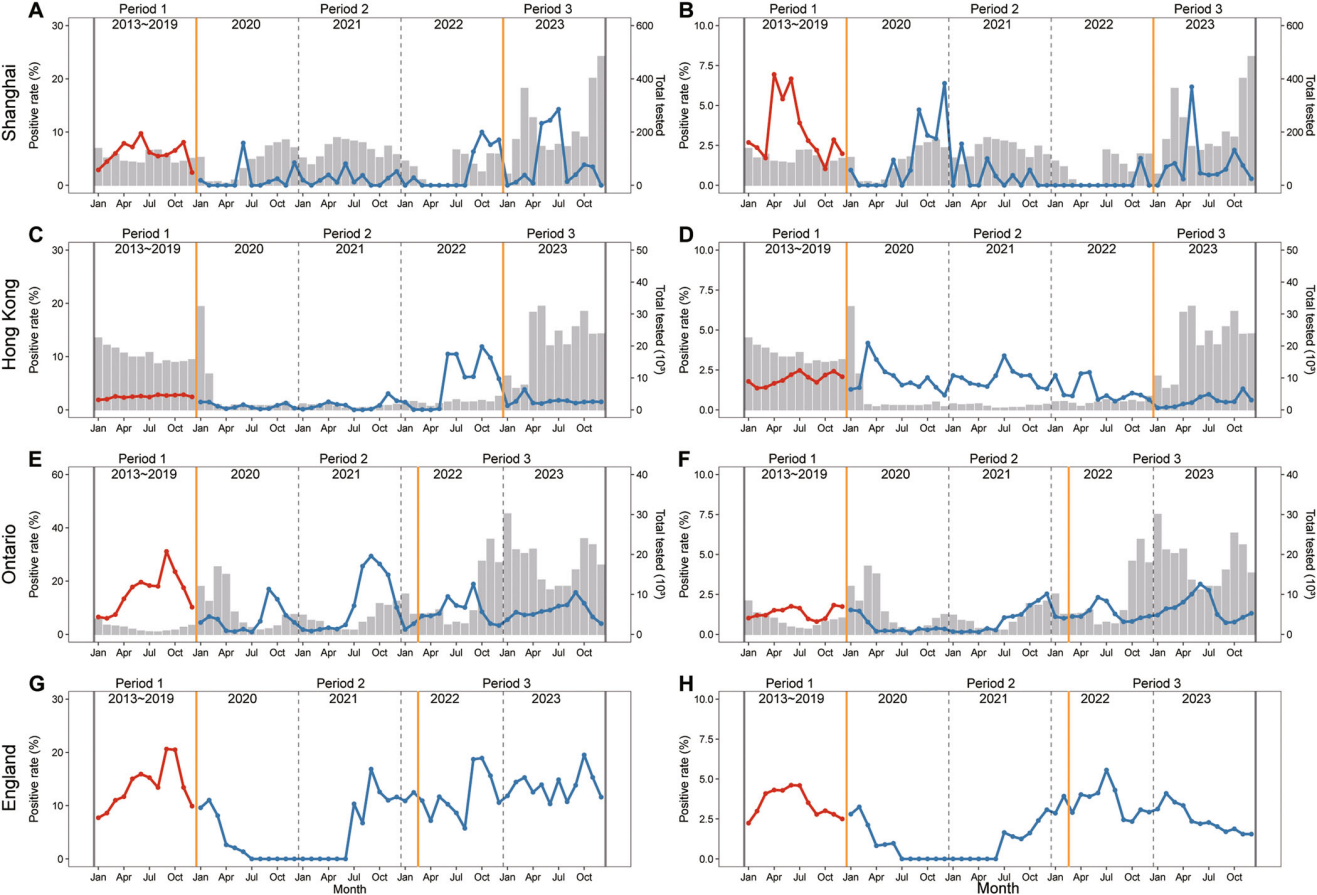
Seasonal pattern of HRV and HAdV in different regions. (A) HRV in Shanghai. (B) HAdV in Shanghai. (C) HRV in Hong Kong. (D) HAdV in Hong Kong. (E) HRV in Ontario. (F) HAdV in Ontario. (G) HRV in England. (H) HAdV in England. The red line indicates the monthly average positive rate during period 1 (pre‐COVID‐19 pandemic, 2013−2019). The blue line represents the monthly positive rate from 2020 to 2023. The gray bar indicates the monthly total number of patients with AIRs tested. The orange solid line delineates the distinct periods for various regions. Due to the COVID‐19 pandemic, from the 8th week of 2020 through the 29th week of 2022, the website of Hong Kong only provided the data of HMPV and HPIV detection of ARI patients under 18 years old. Due to the COVID‐19 pandemic, starting from the 8th week of 2020 through the 29th week of 2022, the official website of Hong Kong exclusively presented data on HRV and HAdV detection among patients under 18 years old with ARIs.

**Figure 4:**
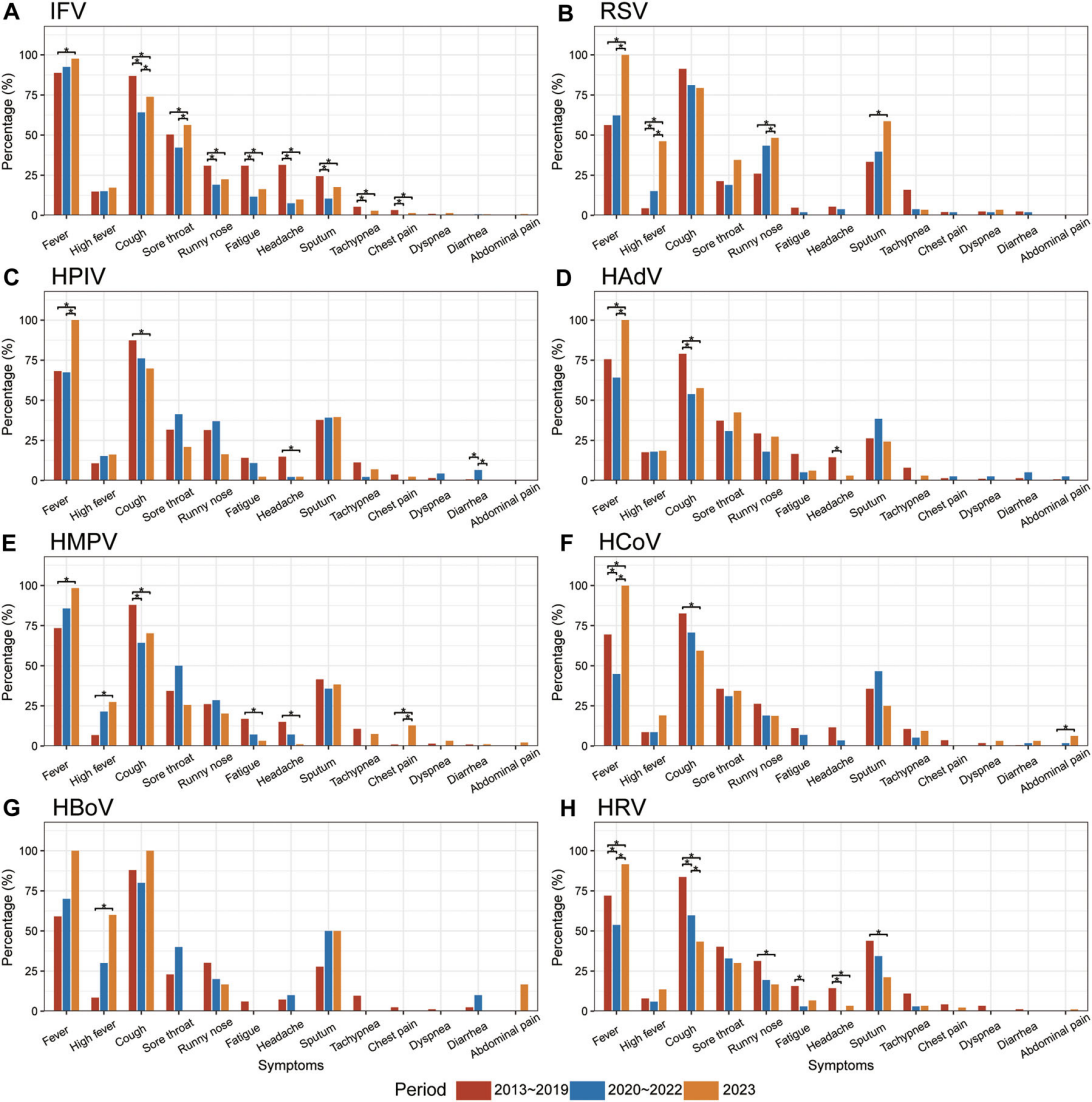
Comparison of clinical symptoms among ARI patients caused by eight viral pathogens across three periods in Shanghai, eastern China. (A) IFV. (B) RSV. (C) HPIV. (D) HAdV. (E) HMPV. (F) HCoV. (G) HBoV. (H) HRV. The colored bars indicate the percentage of clinical symptoms in different periods. Pearson's chi‐square test or Fisher's exact test was conducted to compare the data among the three periods. In case of a significant result, the Scheffe test was used to test the statistical difference between the two periods. *p < 0.05.

By comparing the seasonal patterns of viral infections across different regions globally, it was observed that the impact of the COVID‐19 pandemic or stringent NPIs on the seasonality of various viral pathogens varied. In the case of IFV, there was a 2‒4 month delay in reaching peak positive rates after lifting NPIs in Shanghai, which exhibited a similar pattern to that observed in Hong Kong, Ontario, and England (Figure 1A,C,E,G and Supporting Information S1: Figure S3). The positive detection rate of RSV infection in Shanghai presented a reverse seasonality during period 3 compared to period 1, characterized by peaks occurring in the summer months (Figure 1B). The same pattern was observed in England; however, it did not appear in the other two regions (Figure 1D,F,H and Supporting Information S1: Figure S3). Similarly, a reverse seasonal pattern of HMPV was observed in Shanghai and Hong Kong in period 3, while it exhibited less prominence in Ontario and England (Figure 2 and Supporting Information S1: Figure S4). The seasonal patterns of HPIV, HRV, and HAdV in Shanghai remained relatively stable, consistent with the observed patterns in Hong Kong, Ontario, and England. However, there was a shorter duration of the peak positive rate in period 3 (Figures 3 and 4 and Supporting Information S1: Figures S4 and S5). The seasonal pattern of HCoV remained relatively stable, while the reverse seasonal pattern of HBoV in period 3 was also observed in Shanghai. However, due to the lack of data from other regions, this finding could not be substantiated (Supporting Information S1: Figures S6 and S7).

### 3.3 Clinical Symptoms of Patients With ARI in Shanghai

By comparing the differences in symptoms across various viral infections during the three periods, a significant increase in the proportion of fever or high fever was observed among patients infected with any of the eight viruses after the relaxation of NPIs. Additionally, there was a decrease in the proportion of non‐fever symptoms among ARI patients for almost all viral infections. However, it is noteworthy that certain specific non‐fever symptoms such as sore throat in patients infected with IFV, runny nose and cough with sputum production in patients infected with RSV, and chest pain in patients infected with HMPV demonstrated a distinct and significant rising trend after the lifting of NPIs (Figure 4). Further age‐specific analyses revealed inconsistent changes in the clinical symptoms of patients with ARIs. While there was a significant increase in the proportion of fever or high fever across all age groups, the alterations in non‐fever symptoms were not uniform. Among adults and older individuals, the proportion of non‐fever symptoms decreased for various viral infections such as IFV, HCoV, and HRV infections. Conversely, among children, an increasing trend was observed in non‐fever symptoms for RSV, HCoV, and HRV infections (Supporting Information S1: Figures S8, S9, and 10).

### 3.4 Positive Rates and Coinfection of Viral Infections

**Figure 5:**
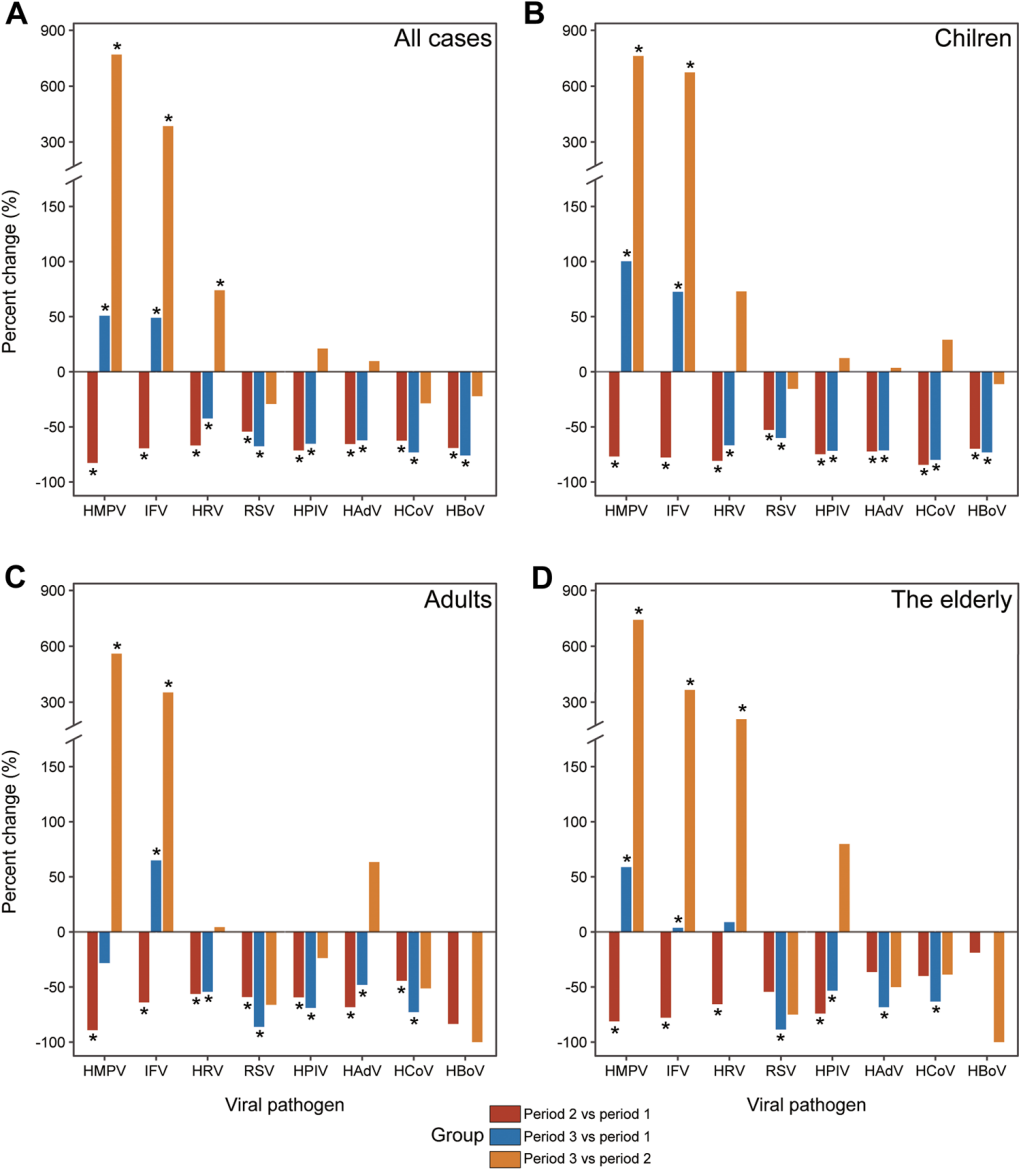
Percentage change in the detection positive rate stratified by age group during three periods in Shanghai, eastern China. (A) All cases. (B) Children. (C) Adults. (D) The elderly. The red, blue, and orange bars indicate positive and negative percent changes for period 2 versus period 1 comparison, period 3 versus period 1 comparison, and period 3 versus period 2 comparison, respectively. Statistically significant changes are denoted with asterisks.

The total number of ARI patients tested for all eight viral pathogens was 15 014 out of 15 134 (99.21%), with 4690 patients (31.24%) testing positive for at least one virus. By comparing the percentage change in positive rates of viral pathogen infections across different periods, it was observed that compared to period 1, both HMPV and IFV exhibited contrasting trends in period 2 and period 3, with the positive rates in period 3 showing an increase compared to period 2. Similar changes were also noted across all age groups. The percent change in the positive rate of HRV showed a decrease in period 2 and period 3 compared to period 1, while the positive rates increased in period 3 compared to period 2. The positive rates of the other five viral pathogens displayed analogous patterns, indicating a decline from period 1 to both period 2 and period 3 (Figure 5). The results of the JPRs for IFV indicated that a singular turn point was observed within the 25−30 age group during periods 1 and 3, while it shifted to the 15−20 age group during period 2 (Supporting Information S1: Figure S11). By examining coinfections between viral pathogens, it was revealed that HCoV exhibited a higher propensity to coinfect patients with other pathogens. Furthermore, compared to the elderly and adults, children had a higher prevalence of coinfections involving multiple viral infections (Supporting Information S1: Figure S13). While comparing the positive rates of eight viral pathogens between ICU and non‐ICU patients, significantly higher positive rates were observed for all viral pathogens except IFV in ICU patients (Supporting Information S1: Figure S12A). Interestingly, among children, the positive rates of all viral pathogens except IFV were higher in ICU patients compared to non‐ICU patients. However, only the positive rate of IFV was significantly higher in adult non‐ICU patients than in ICU; this difference was not statistically significant among older individuals (Supporting Information S1: Figure S13B–D).

### 3.5 Viral Pathogen Spectrum

**Figure 6:**
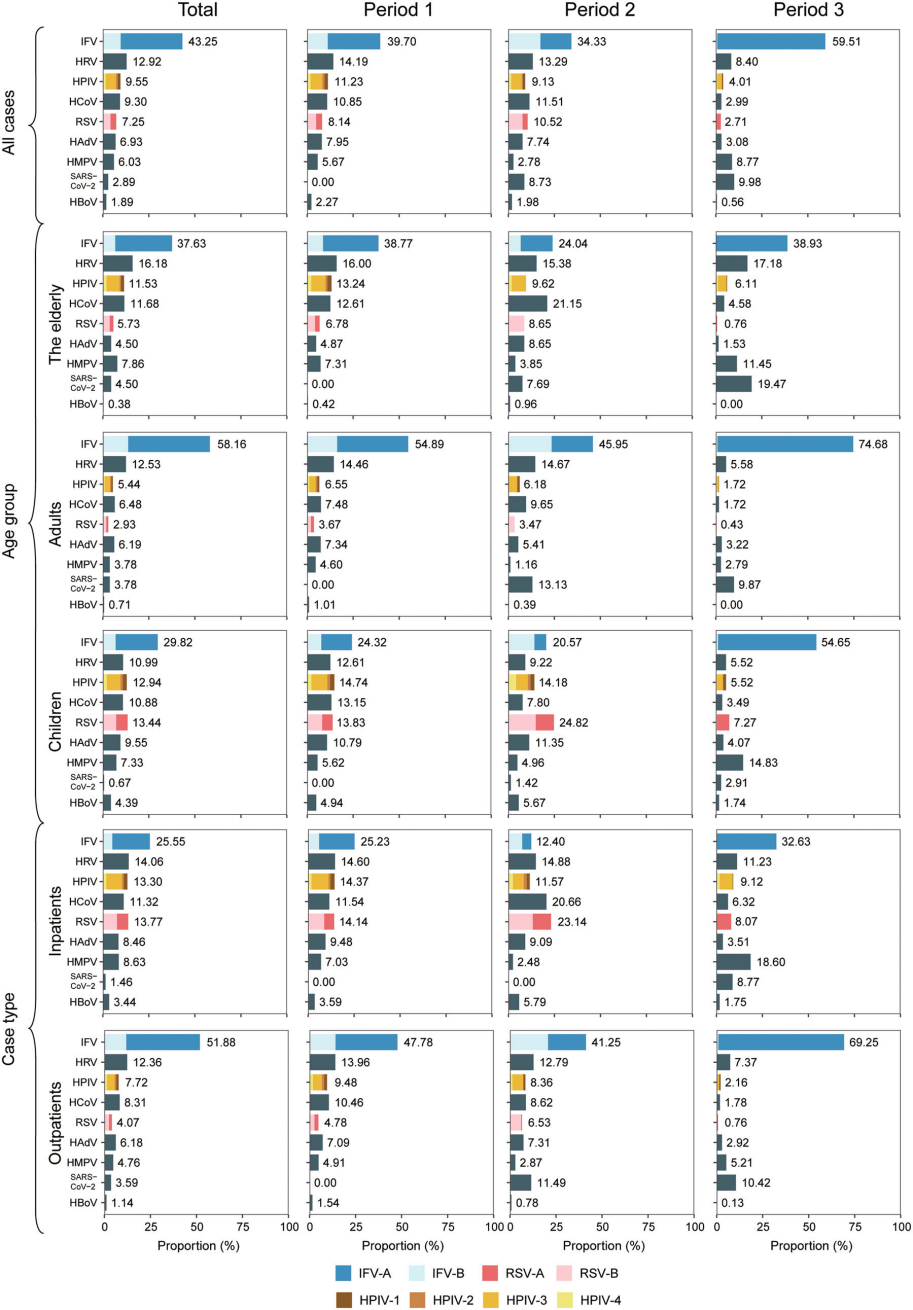
Viral composition of patients with ARIs in Shanghai, eastern China. A total of 15 134 ARIs patients were tested for all eight viral pathogens. The length of colored bars and the numbers behind them indicate the proportion of each pathogen, calculated by dividing its positive count by the total positive count across all pathogens. For IFV, RSV, and HPIV, the subtypes are indicated by different colors on each bar. SARS‐CoV‐2 detection began in July 2020 and is also included in this figure.

According to the proportion of positive detections for various viruses, IFV was consistently identified as the most frequently detected viral pathogen throughout the entire study period, accounting for 43.25% of all positive detections. It was followed by HRV at 12.92%, HPIV at 9.55%, HCoV at 9.30%, RSV at 7.25%, HAdV at 6.93%, HMPV at 6.03%, SARS‐CoV‐2 at 2.89%, and HBoV at 1.89%. Further genotyping analysis of IFV revealed that IFV‐A played a predominant role, accounting for 77.70% of all IFV‐infected patients (2260 cases). Among these cases, H3N2 and H1N1 accounted for 48.10% (1087/2260) and 21.02% (475/2260), respectively, while untyped IFV‐A accounted for 8.58% (194/2260). On the other hand, IFV‐B only accounted for 22.30%, with Victoria and Yamagata stains accounting for 7.70% (174/2260) and 3.23% (73/2260), respectively, while untyped IFV‐B accounted for 11.37% (257/2260). Among HPIVs, the majority proportion was constituted by HPIV‐3 (58.32%), followed by HPIV‐4 (16.63%), HPIV‐1 (15.63%), and HPIV‐2 (9.42%). Among patients infected with RSV, RSV‐B emerged as the predominant genotype, accounting for 57.26%, while RSV‐A constituted 42.74%. Further analysis based on age groups revealed that IFV was the predominant pathogen across all age groups, followed by HRV in elderly populations (16.18%) and adults (12.53%), while RSV ranked second among children with a proportion of 13.44%. The ranking of other viruses varied among different age groups (Figure 6).

Further period‐specific analyses revealed significant shifts in the ranking of viral pathogens for each age group in periods 2 and 3, as compared to period 1. In period 1, the ranking of viral pathogens within each age group generally aligned with the results of the entire study period. However, a notable change occurred in period 2 where IFV‐B emerged as the predominant genotype of IFV, especially among children and adults, while this trend was not observed among the elderly individuals. Another noteworthy change during this period was the increased proportion of RSV, which exhibited a rise from 13.83% to 24.82% among children. In period 3, there was a significant surge in the proportion of IFV (59.51%) compared to periods 1 and 2; moreover, IFV‐A (98.75%) became nearly exclusive across all age groups. Simultaneously, the proportion of HMPV demonstrated a substantial increase in children and elderly populations, while SARS‐CoV‐2 emerged as the second most prevalent virus among adults and elderly individuals (Figure 6).

The ranking of the viral pathogen spectrum differed between inpatients and outpatients. With the exception of IFV and SARS‐CoV‐2, the proportion of seven viruses was higher among inpatients compared to outpatients. Among inpatients, the top five viruses were IFV, HRV, RSV, HPIV, and HCoV; whereas in outpatients, the top five viruses were IFV, HRV, HCoV, HPIV, and HAdV. Notably, RSV had a significantly higher proportion among inpatients than outpatients (13.77% vs. 4.07%, *p *< 0.001). Comparing period 1 to period 2 among inpatients revealed that RSV (23.14%) and HCoV (20.66%) emerged as the two most prevalent viruses; however, during period 3, IFV re‐emerged as the predominant virus with a proportion of 32.63%, followed by HMPV (18.60%), HRV (11.23%), HPIV (9.12%), and SARS‐CoV‐2 (8.77%). Among outpatients across all age groups during all periods examined, IFV consistently remained the most frequently detected viral pathogen, while SARS‐CoV‐2 rose to third place during period 2 and second place during period 3 (Figure 6).

## 4 Discussion

The lifting of stringent NPIs was found to be accompanied by alterations in both the positive rates and seasonal patterns of respiratory viral pathogens among ARI patients in this study. Moreover, the clinical symptoms presented by patients with ARIs were influenced following the relaxation of stringent NPIs, leading to a significant shift in the spectrum of viral pathogens among these individuals. Notably, Shanghai has observed a remarkably low positive rate for non‐SARS‐CoV‐2 respiratory viruses during the COVID‐19 pandemic, which can potentially be attributed to the implementation of NPIs [5, 11, 16]. To effectively mitigate the transmission of SARS‐CoV‐2, the China government rigorously implemented NPIs such as mandatory mask‐wearing, stringent social distancing measures, closure of schools or workplaces when necessary, and travel restrictions [17]. The implementation of these restrictive measures has the potential to disrupt the epidemiology of other respiratory viruses that share similar transmission routes, as observed in previous instances in different countries [18, 19, 20]. Maintaining a low prevalence compared to regular seasonal epidemics may initially appear advantageous; however, there is mounting evidence that we need to pay attention to the potential of immunity debt, which refers to an increased susceptibility of large populations to a specific disease following prolonged periods of reduced exposure [21]. In fact, numerous countries have observed a resurgence of respiratory pathogens following the relaxation of most NPIs at the beginning of 2022. For instance, starting from April 2022 in Australia, influenza cases have exceeded the average levels recorded over the past 5 years [22]. The findings from our study align with the occurrence of immunity debt, as evidenced by the resurgence of IFV and HMPV in 2023. Furthermore, it is foreseeable that reduced vaccination against other respiratory pathogens during the COVID‐19 pandemic, along with changes in human activity patterns, may contribute to an increased risk of infection [23].

The current study unveiled a significant rise in the occurrence of fever or high fever among patients with ARIs during the post‐COVID‐19 pandemic, potentially attributed to the enduring impact of SARS‐CoV‐2 infection on immune system functionality and the nature of persistent molecular and cellular changes. Previous studies have indicated that following SARS‐CoV‐2 infection, levels of various inflammatory cytokines such as IL‐6, IL‐2, and TNF‐α can remain elevated to differing extents for an extended period, which may facilitate inflammation development and break through the blood−brain barrier to affect thermoregulatory function. Consequently, individuals infected with respiratory viral pathogens are more susceptible to experiencing fever [24, 25]. These findings underscore the importance of prioritizing health management after the COVID‐19 pandemic.

Another notable finding was the occurrence of an off‐season epidemic and a delayed surge in viral pathogens (IFV, RSV, HMPV, and HBoV) in Shanghai following the lifting of stringent NPIs. This pattern was also observed in several countries for certain respiratory pathogens. For example, in the Netherlands, there was a higher positive rate of RSV infection reported during the summers of 2021 and 2022 [26]. Numerous studies have suggested that this pattern is linked to the relaxation of NPIs, particularly the reopening of borders and schools [26, 27]. Additionally, our findings indicate that the impact of NPIs on the seasonality of viral pathogens in Shanghai and Hong Kong is more pronounced compared to Ontario and England, suggesting that implementation of stricter NPIs may lead to more substantial alterations in the seasonal patterns of these pathogens. Interestingly, there was considerable heterogeneity observed in epidemic patterns across countries and regions; however, regions with geographic proximity and similar NPIs may experience comparable patterns (e.g., the epidemic patterns of IFV in Shanghai and Hong Kong). This observed heterogeneity cannot be attributed to the lifting of NPIs, as differences in geographic location and corresponding variations in social and physical environments might also contribute to this variability [27].

In addition, influenza B emerged as the predominant stain in Shanghai during the period from 2020 to 2022; however, subsequent to the lifting of NPIs, influenza A regained its dominance in the following influenza season (Figure 5). The extensive documentation by numerous researchers has highlighted the prevalence of Influenza B in China during the COVID‐19 pandemic. For instance, in Sichuan and Guangdong provinces, the proportion of influenza B exceeded 99% during the 2020−2021 influenza season [28, 29]. The re‐emergence of influenza A as the dominant type after the lifting of NPIs can be attributed to two factors. First, a decline in natural and artificial immunity during the COVID‐19 pandemic may have increased susceptibility. Second, compared to influenza B, influence A tends to exhibit higher transmissibility [10]. Consequently, with NPIs being lifted and international travel resuming, these factors particularly contribute to facilitating the global spread of influenza A. In our analysis of the viral pathogen spectrum, we identified RSV‐B as the predominant genotype among RSV infections, a finding consistent with prior research [30]. This contrasts with observations from other regions in China and internationally, such as Hangzhou, China, and Chicago, USA, where RSV‐A is more commonly the dominant genotype [31, 32]. The disparity in predominant genotypes could be attributed to multiple factors. One possibility is that RSV infections are frequently diagnosed in children, whereas they may go undetected in adults due to milder symptoms. Therefore, the relatively low number of RSV‐positive cases in our data set might also contribute to this difference. Notably, despite the variation in the predominant subtypes, a similar reverse seasonal pattern of RSV was observed across different regions. This reverse seasonal pattern suggests that NPIs have significantly shaped the prevalence dynamics of RSV.

Changes in the epidemiologic pattern of respiratory diseases have historically occurred following pandemics, such as the 1918 influenza pandemic and the 2009 H1N1 pandemic [33]. Some researchers have posited that these disruptions in seasonal patterns would typically be confined to the initial year of a pandemic pathogen's circulation, gradually converging toward a normal pattern in subsequent years [34]. The COVID‐19 pandemic has significantly altered population contact and mobility patterns, thereby impacting the global seasonal cycle of respiratory diseases. Consequently, it remains uncertain whether non‐SARS‐CoV‐2 respiratory viral pathogens will revert back to their customary seasonal cycle over time.

### 4.1 Limitations

The limitations of this study should be acknowledged. First, data collection was limited to patients with ARIs from 12 hospitals in Shanghai, eastern China, potentially introducing selection bias. Conducting a larger multicenter research trial would greatly contribute to the control, prevention, and treatment of respiratory viral infections in the future. Second, we did not analyze the strength of NPI measures across countries, which may have led to potential inaccuracies within our findings. Considering the global variation in response to the COVID‐19 pandemic and regions, it is crucial to encourage similar studies worldwide for more effective management of respiratory infections post‐COVID‐19 pandemic. In addition, the surveillance data generated in other global regions may differ in terms of study population characteristics (such as age group, immunization background, and case definition), study settings (including community, outpatient, inpatient and ICU), the number of etiological agents investigated, and laboratory methods used (PCR/serological tests/culture). These differences could potentially impact the study results.

## 5 Conclusions

In conclusion, this study investigated the patterns of eight respiratory viral infections in Shanghai in comparison to multiple other global regions in the context of the COVID‐19 pandemic. These findings provide valuable insights into the prevention and control of prevalent respiratory viral pathogens following the lifting of NPIs, as well as the administration of antivirals or vaccines. Furthermore, these findings enhance our evidence‐based knowledge for developing targeted policies and measures in preparation for future global pandemics.

## Author Contributions

Li‐Qun Fang, Zhongjie Li, and Lipeng Hao conceived and designed the study. Chuchu Ye, Yao Tian, Li Zhang, Bing Zhao, and Yifeng Shen collected and checked the data. Chuchu Ye, Yao Tian, Dazhu Huo, Ting Zhang, Xinli Jiang, Xuancheng Hu, and Haiyang Zhang analyzed and interpreted the data. Chuchu Ye, Yao Tian, and Dazhu Huo wrote the first draft of the manuscript. Li‐Qun Fang, Zhongjie Li, and Lipeng Hao revised and supervised the paper. All authors approved the final version for submission and agreed to be accountable for all aspects of the work.

## Ethics Statement

The surveillance protocol underwent a comprehensive review and received approval from the ethics review committee of the Shanghai Pudong CDC (Approval Number: 20230605‐001).

## Conflicts of Interest

The authors declare no conflicts of interest.

## Supporting information

Supporting information.

## Data Availability

The data sets used and/or analyzed during the current study are available from the corresponding author upon reasonable request.
