# Supplementary material for: Changes in Epidemics of Respiratory Viral Infections Resulted From the COVID‐19 Pandemic in Shanghai
Source: J Med Virol. 2024 Nov 8;96(11):e70034. doi: 10.1002/jmv.70034 (PMC11600481; doi:10.1002/jmv.70034)
Supplement: Supplementary file 1 — Supporting information. [file JMV-96-e70034-s001.docx]

**Supplementary appendix**

Changes in epidemics of respiratory viral infections resulted from the COVID-19 pandemic in Shanghai

**Table of Contents**

| **Page** | **Item** |
| --- | --- |
| 1 | **Supplementary Figure 1. The flow diagram of data included in this study.** |
| 2 | **Supplementary Figure 2. The Oxford stringency index in different regions.** |
| 3 | **Supplementary Figure 3. Seasonal fitted curves of IFV and RSV in different regions.** |
| 4 | **Supplementary Figure 4. Seasonal fitted curves of HMPV and HPIV in different regions.** |
| 5 | **Supplementary Figure 5. Seasonal fitted curves of HRV and HAdV in different regions.** |
| 6 | **Supplementary Figure 6. Seasonal pattern of HCoV and HBoV in Shanghai, eastern China.** |
| 7 | **Supplementary Figure 7. Seasonal pattern of HCoV and HBoV in Shanghai, eastern China.** |
| 8 | **Supplementary Figure 8. Comparison of clinical symptoms (the elderly) in eight viral pathogens between three periods in Shanghai, eastern China.** |
| 9 | **Supplementary Figure 9. Comparison of clinical symptoms (adults) in eight viral pathogens between three periods in Shanghai, eastern China.** |
| 10 | **Supplementary Figure 10. Comparison of clinical symptoms (children) in eight viral pathogens between three periods in Shanghai, eastern China.** |
| 11 | **Supplementary Figure 11. The Join-Point regression of the positive rates of IFV by age of ARIs patient in Shanghai, eastern China.** |
| 12 | **Supplementary Figure 12. Comparison of positive rates for eight viral pathogens in ICU and non-ICU patients in Shanghai, eastern China.** |
| 13 | **Supplementary Figure 13. The co-infection pattern of ARIs patients by age group in Shanghai, eastern China.** |

**Supplementary Figure 1. The flow diagram of data included in this study.**

**
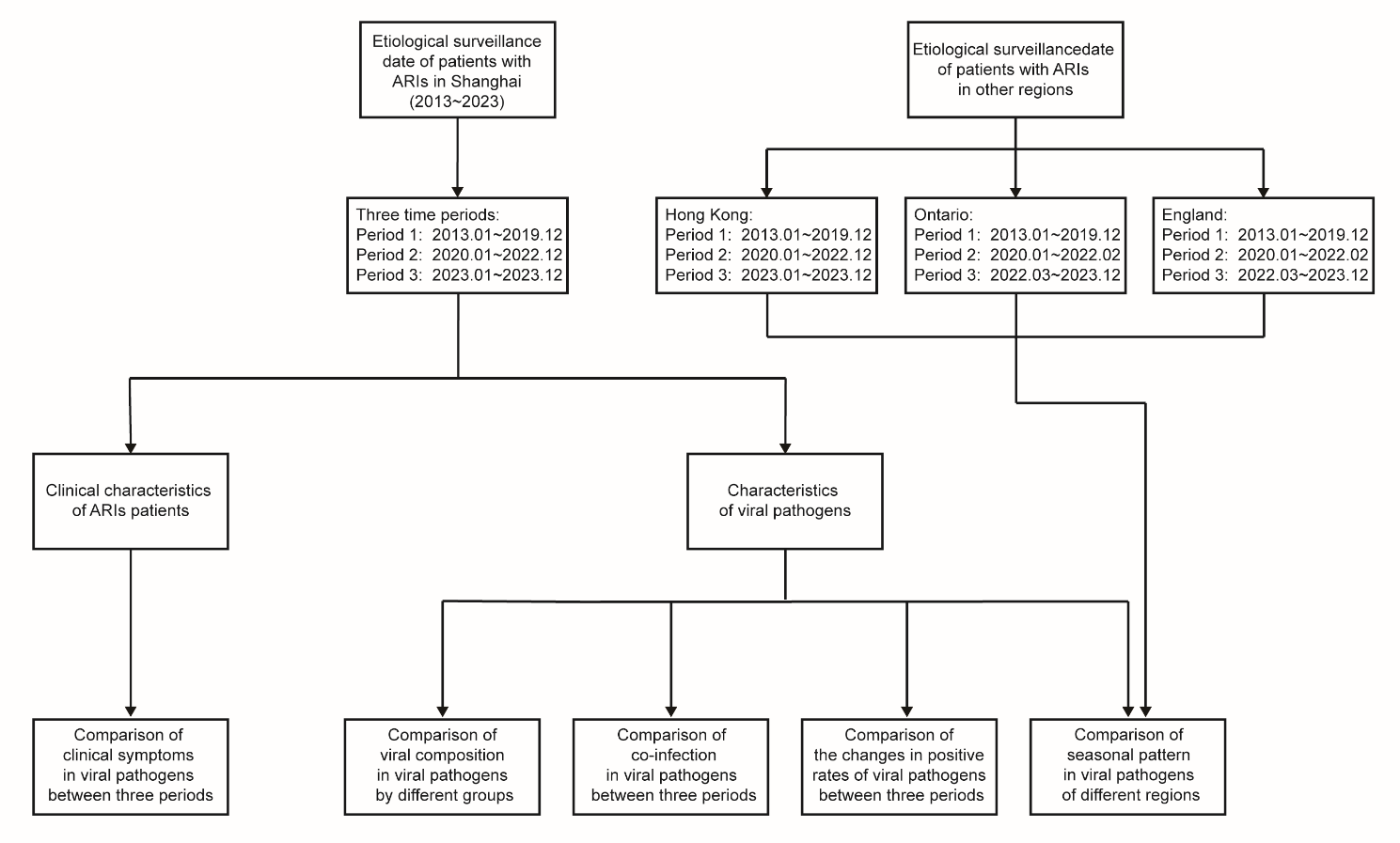
**

**Supplementary Figure 2. The Oxford** **stringency index in different regions.** (A) China. (B) Hong Kong. (C) Canada. (D) United Kingdom. The blue line indicates the daily stringency index in different regions. The orange line indicates the time to lifting the NPIs in different regions. The red line indicates the stringency index equal to 40.

**
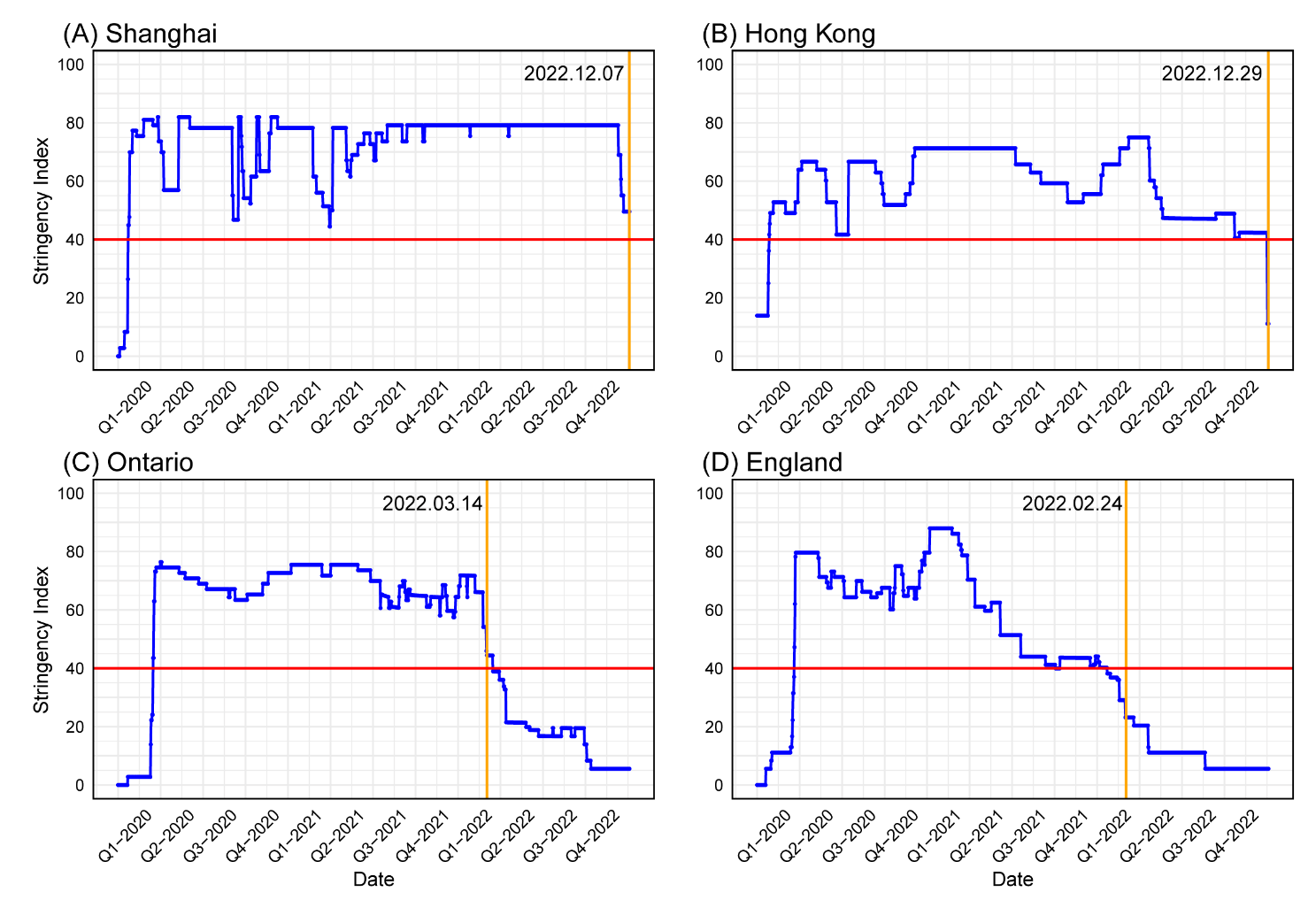
**

**Supplementary Figure 3. Seasonal fitted curves of IFV and RSV in different regions.** (A) IFV in Shanghai. (B) RSV in Shanghai. (C) IFV in Hong Kong. (D) RSV in Hong Kong. (E) IFV in Ontario. (F) RSV in Ontario. (G) IFV in England. (H) RSV in England. The colored bold lines indicate the fitted curves for different periods. The colored slim lines represent the monthly positive rates for different periods. The black dashed lines represent the seasonal trends calculated by seasonal decomposition. The fitted curves are fitted by locally weighted smoothing method (LOESS).

**
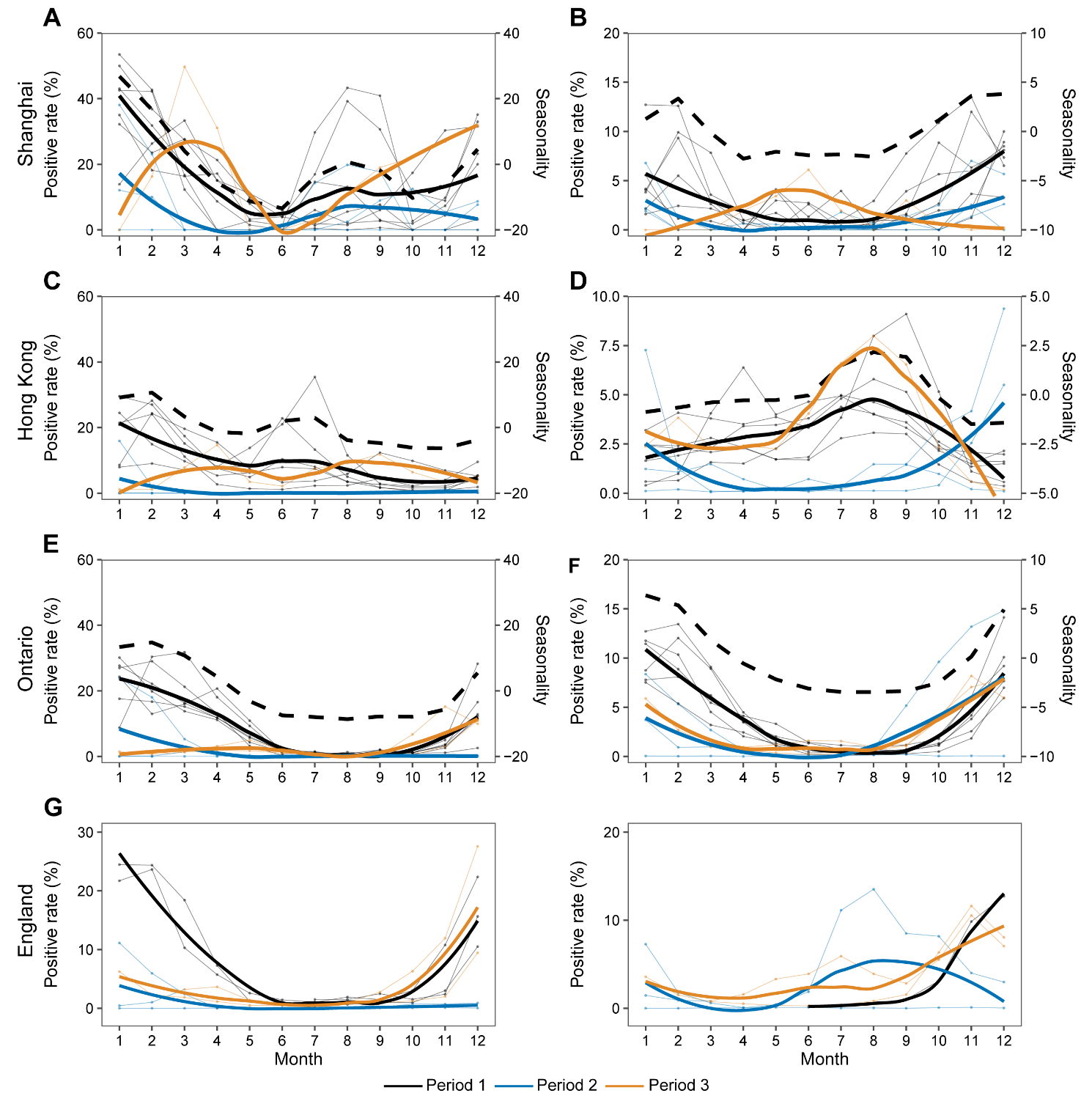
**

**Supplementary Figure 4. Seasonal fitted curves of HMPV and HPIV in different regions.** (A) HMPV in Shanghai. (B) HPIV in Shanghai. (C) HMPV in Hong Kong. (D) HPIV in Hong Kong. (E) HMPV in Ontario. (F) HPIV in Ontario. (G) HMPV in England. (H) HPIV in England. The colored bold lines indicate the fitted curves for different periods. The colored slim lines represent the monthly positive rates for different periods. The black dashed lines represent the seasonal trends calculated by seasonal decomposition. The fitted curves are fitted by LOESS.**
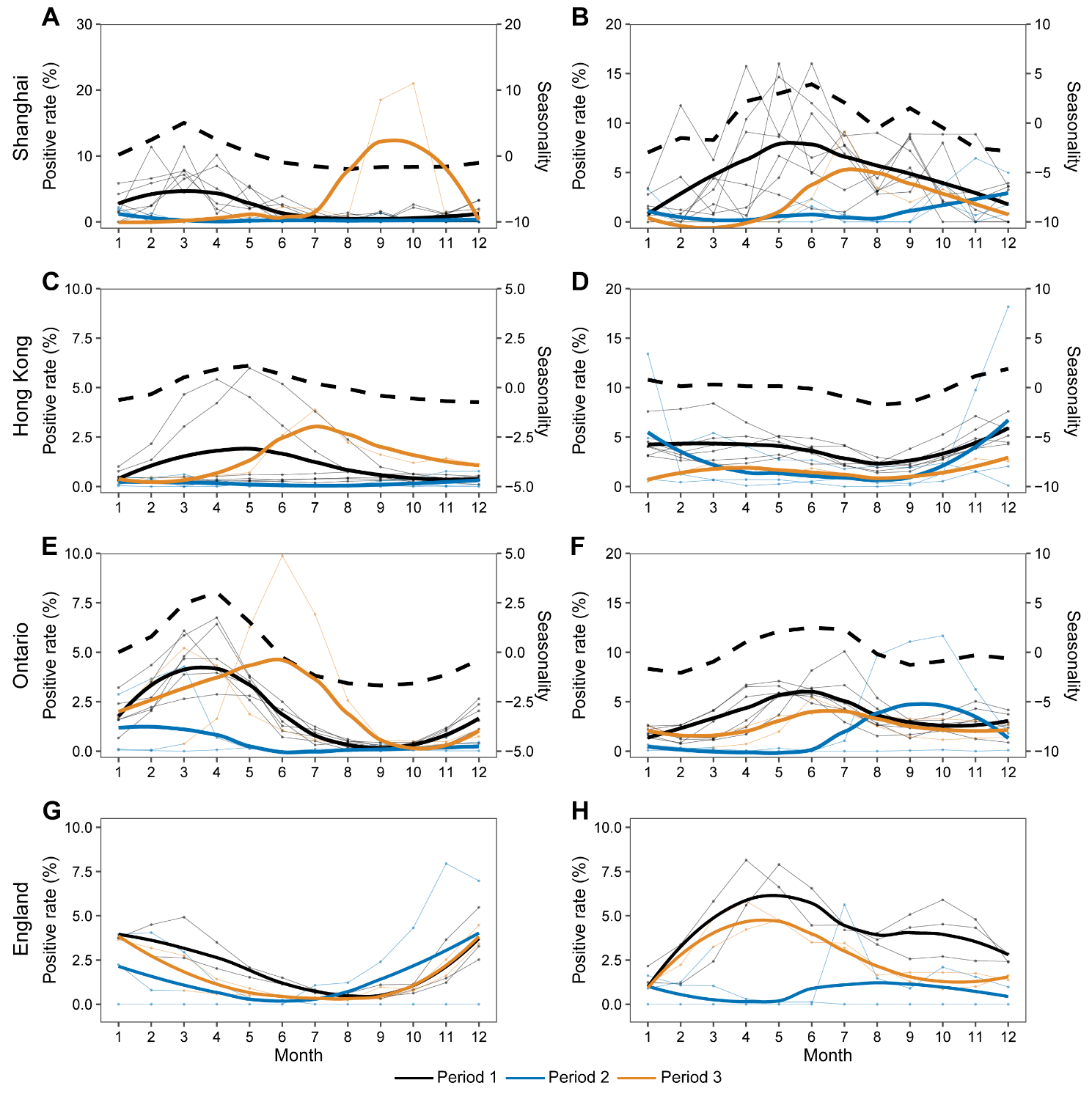
**

**Supplementary Figure 5. Seasonal fitted curves of HRV and HAdV in different regions.** (A) HRV in Shanghai. (B) HAdV in Shanghai. (C) HRV in Hong Kong. (D) HAdV in Hong Kong. (E) HRV in Ontario. (F) HAdV in Ontario. (G) HRV in England. (H) HAdV in England. The colored bold lines indicate the fitted curves for different periods. The colored slim lines represent the monthly positive rates for different periods. The black dashed lines represent the seasonal trends calculated by seasonal decomposition. The fitted curves are fitted by LOESS.**
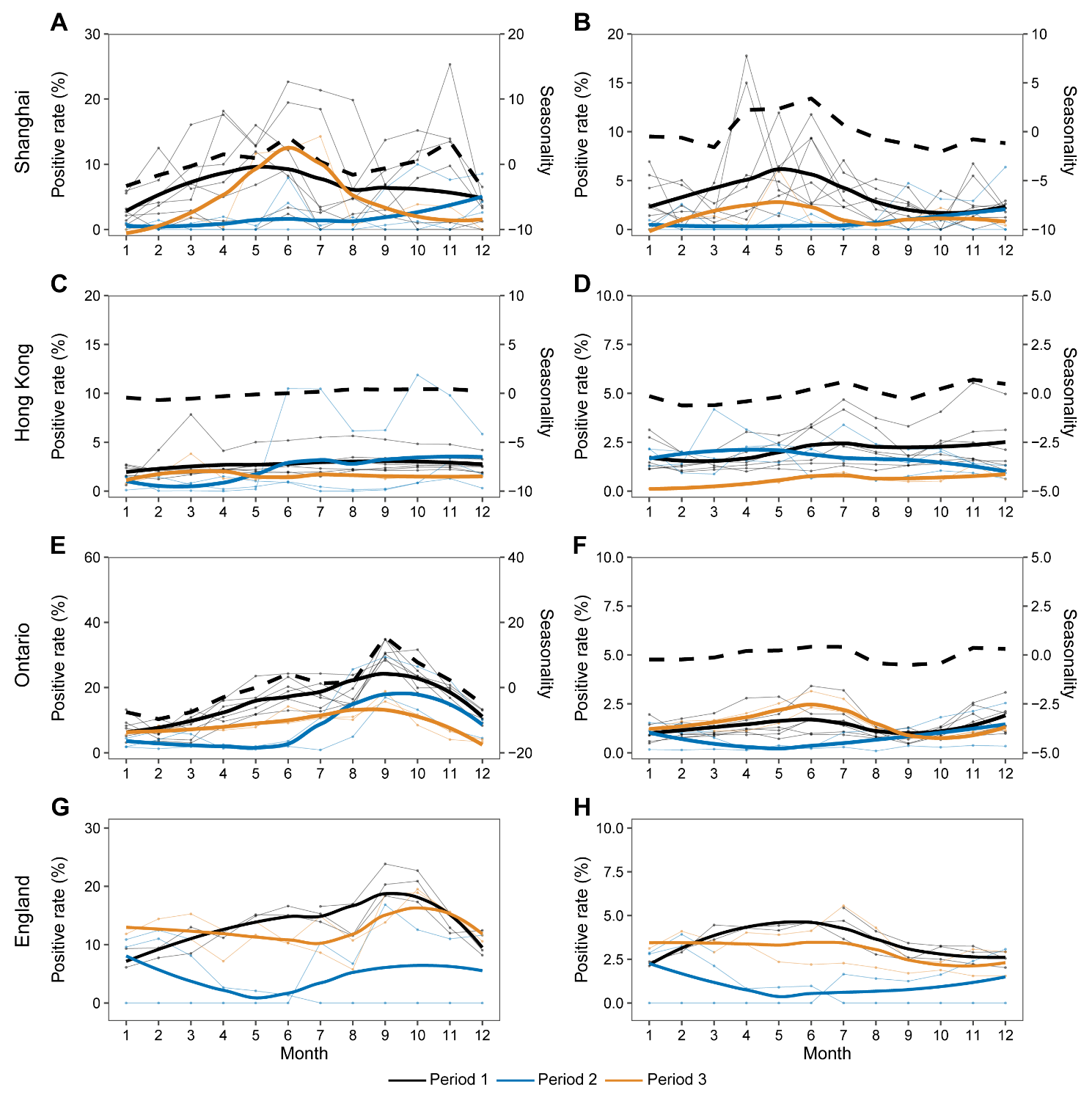
**

**Supplementary Figure 6. Seasonal pattern of HCoV and HBoV in Shanghai, eastern China.** (A) HCoV. (B) HBoV. The red line indicates monthly average positive rate in pre-COVID-19 pandemic. The blue line indicates monthly positive rate between 2020 to 2023. The grey bar indicates the monthly total tested. The orange solid line indicates the different periods. The dashed lines indicate the different years.
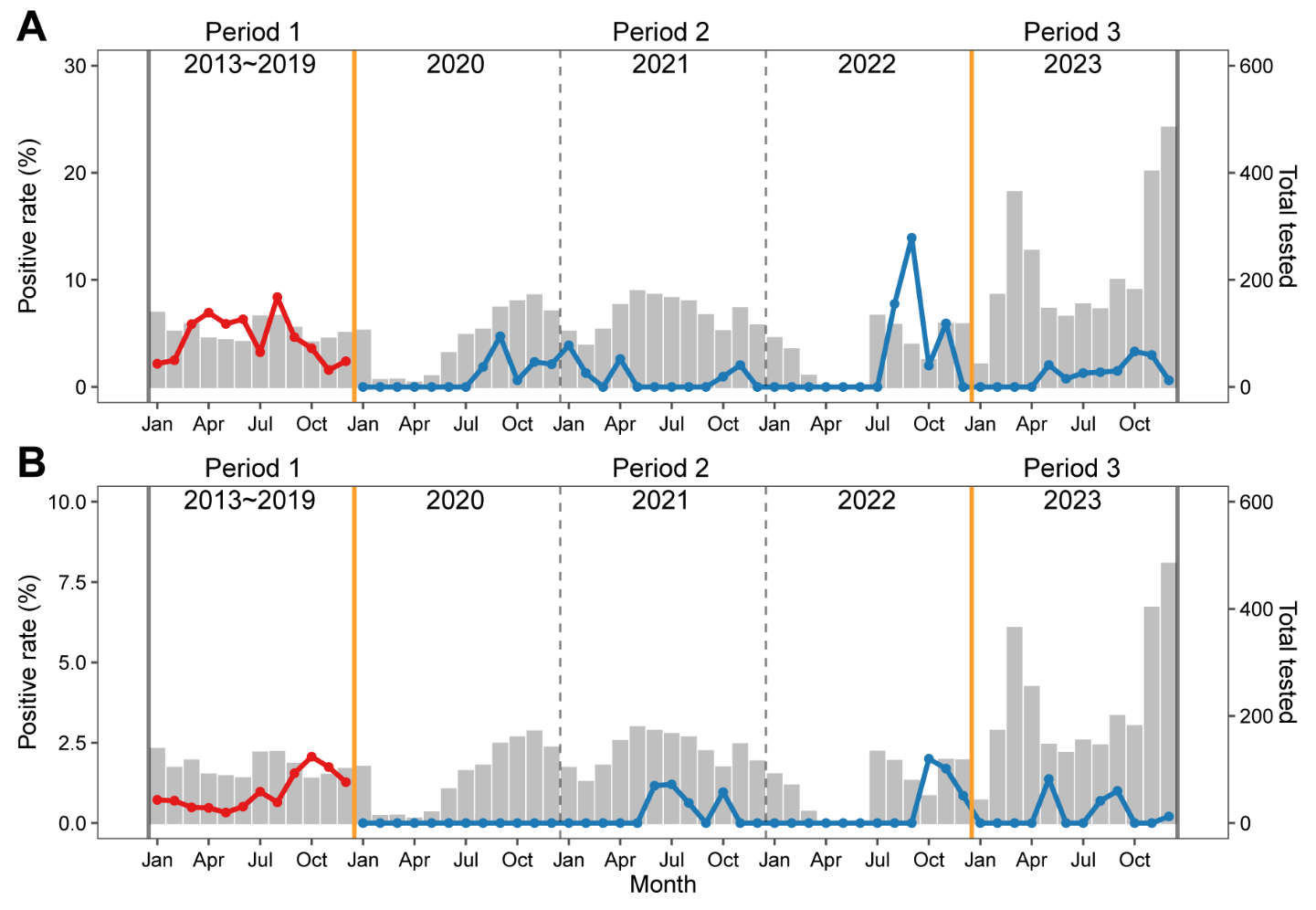


**Supplementary Figure 7. Seasonal pattern of HCoV and HBoV in Shanghai, eastern China.** (A) HCoV. (B) HBoV. The colored bold lines indicate the fitted curves for different periods. The colored slim lines represent the monthly positive rates for different periods. The black dashed lines represent the seasonal trends calculated by seasonal decomposition. The fitted curves are fitted by LOESS.

**
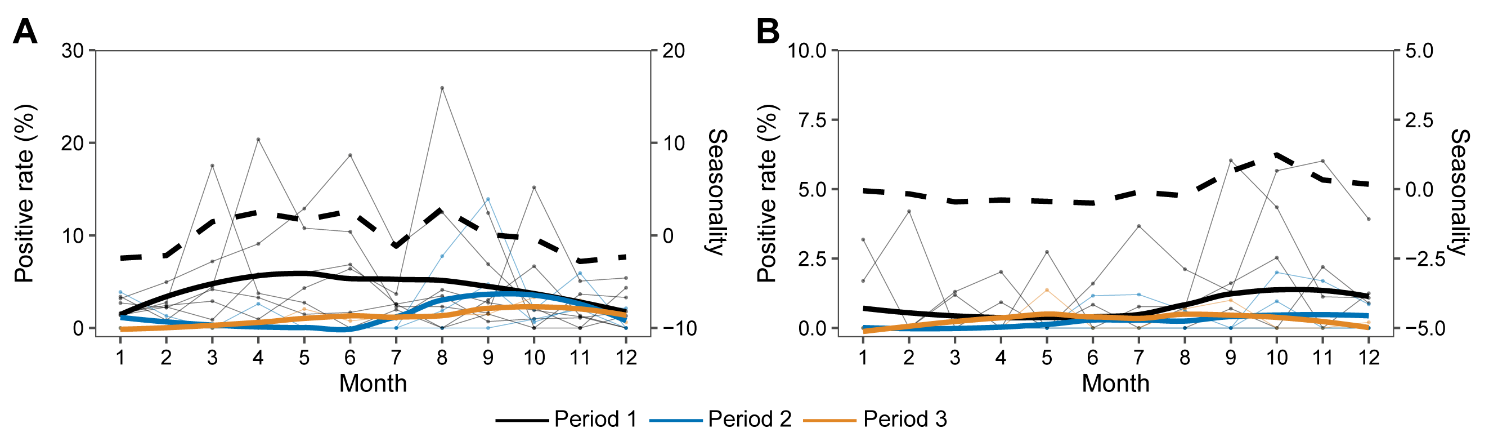
**

**Supplementary Figure 8. Comparison of clinical symptoms (the elderly) in eight viral pathogens between three periods in Shanghai, eastern China.** (A) IFV. (B) RSV. (C) HPIV. (D)HAdV. (E) HMPV. (F) HCoV. (G)HBoV. (H) HRV. The colored bars indicate the percentage of clinical symptoms in different periods. The comparison among three periods was calculated by Pearson’s Chi-square test or Fisher’s exact test. If the test was significant, Scheffe test was used to test the statistical difference. *p values<0.05.

**
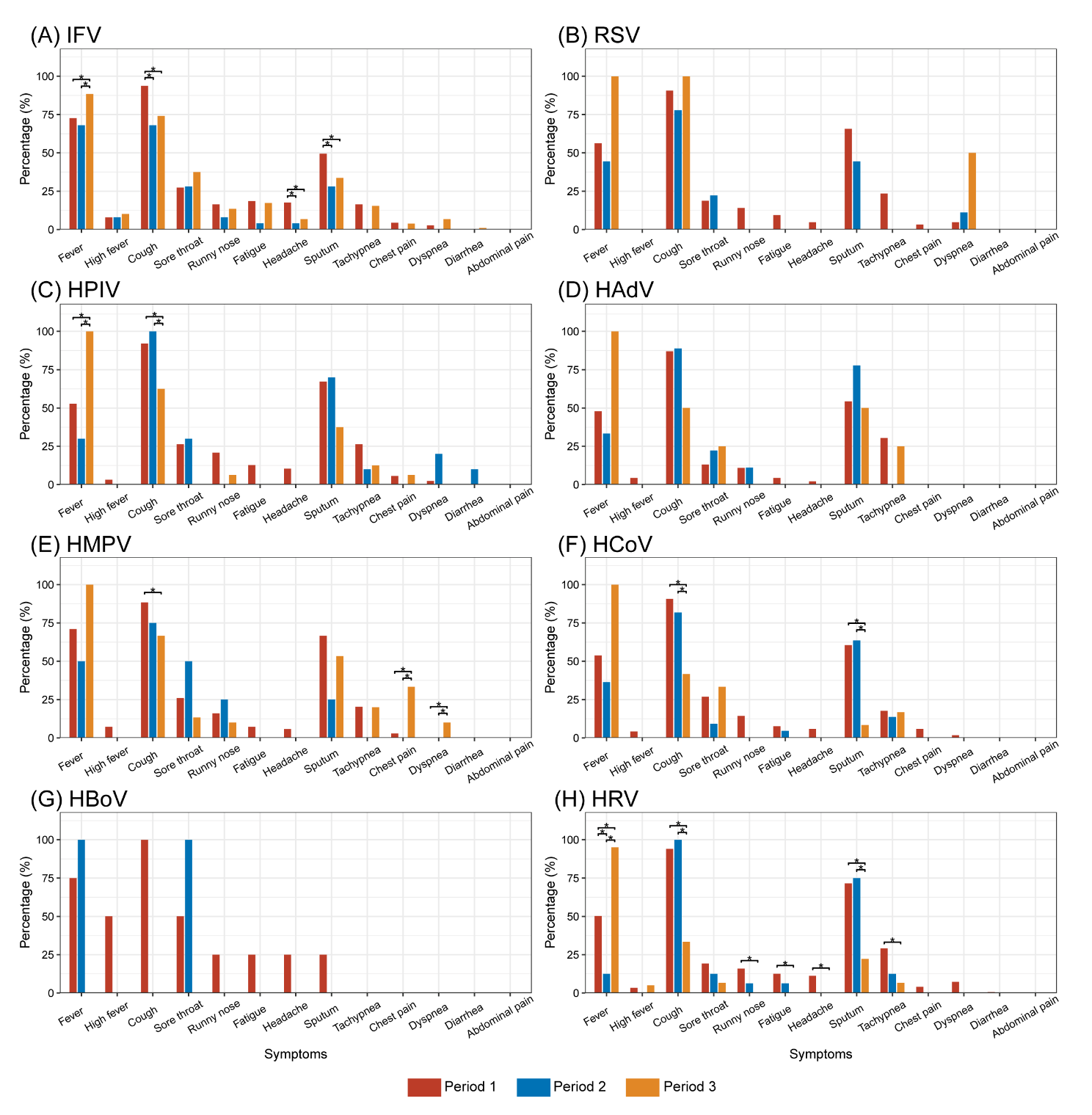
**

**Supplementary Figure 9. Comparison of clinical symptoms (adults) in eight viral pathogens between three periods in Shanghai, eastern China.** (A) IFV. (B) RSV. (C) HPIV. (D)HAdV. (E) HMPV. (F) HCoV. (G)HBoV. (H) HRV. The colored bars indicate the percentage of clinical symptoms in different periods. The comparison among three periods was calculated by Pearson’s Chi-square test or Fisher’s exact test. If the test was significant, Scheffe test was used to test the statistical difference. *p values<0.05.

**
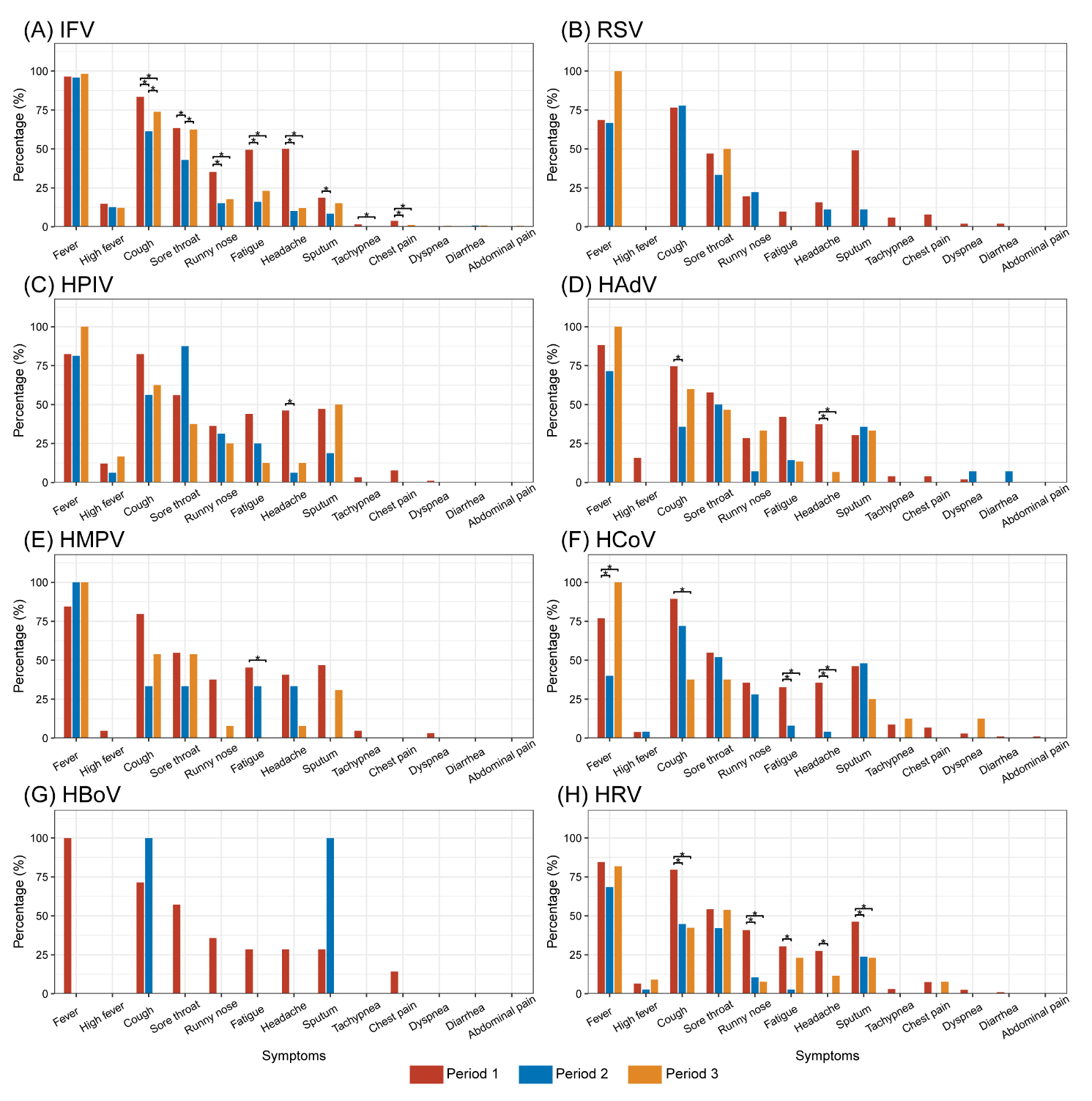
**

**Supplementary Figure 10. Comparison of clinical symptoms (children) in eight viral pathogens between three periods in Shanghai, eastern China.** (A) IFV. (B) RSV. (C) HPIV. (D)HAdV. (E) HMPV. (F) HCoV. (G)HBoV. (H) HRV. The colored bars indicate the percentage of clinical symptoms in different periods. The comparison among three periods was calculated by Pearson’s Chi-square test or Fisher’s exact test. If the test was significant, Scheffe test was used to test the statistical difference. *p values<0.05.

**
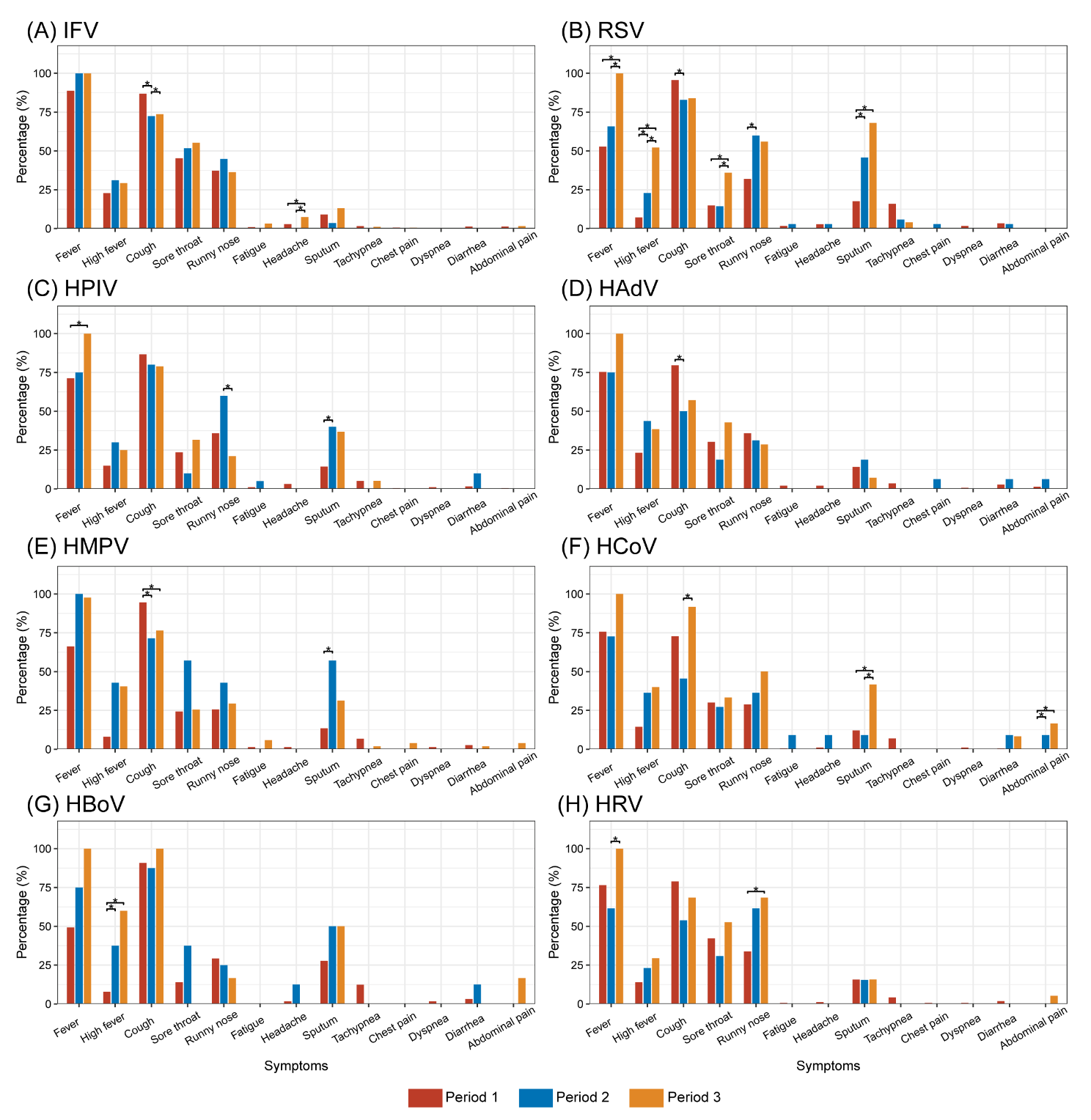
**

**Supplementary Figure 11.** **The Join-Point regression of the positive rates of IFV by age of ARIs patient in Shanghai, eastern China.** The colored dashed lines indicate the positive rate of IFV in different period. The colored solid lines indicate the fitting value of the Join-Point regression.

**
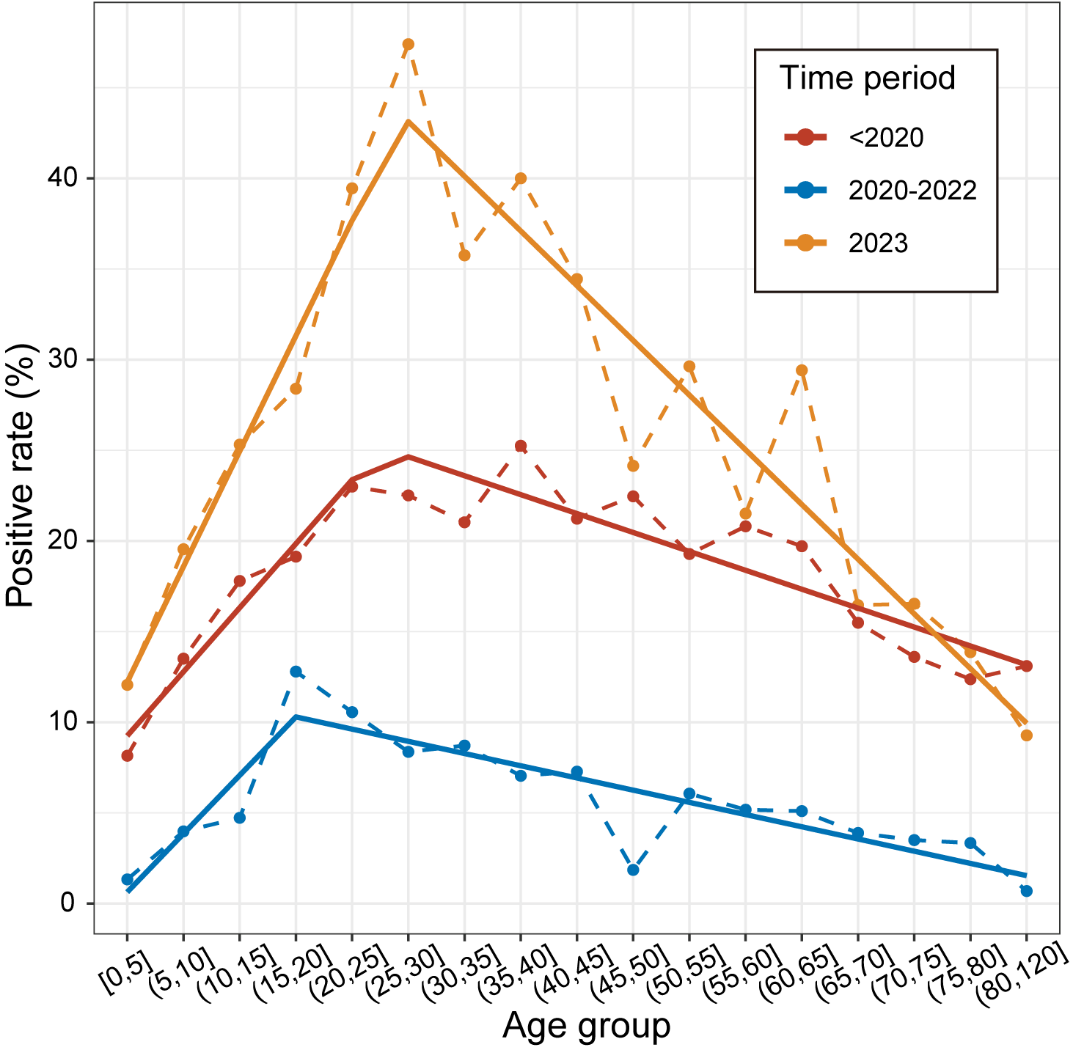
**

**Supplementary Figure 12. Comparison of positive rates for eight viral pathogens in ICU and non-ICU patients in Shanghai, eastern China.** (A) All cases. (B) Children. (C) Adults. (D) The elderly. The positive rate of each pathogen among 15,014 patients (965 ICU patients and 14,049 non-ICU patients) tested for all the eight viral pathogens was compared for different age groups. The red bar indicates the positive rate of ICU patients and the blue bar indicates the positive rate of non-ICU patients. Positive rate was calculated by taking the positive number of each pathogen as the numerator and the number of each viral pathogen tested as denominator. The significant difference of the positive rate (Pearson’s Chi-square test or Fisher’s exact test) is indicated. IFV: influenza virus. RSV: respiratory syncytial virus. HRV: human rhinovirus. HPIV: human parainfluenza. HCoV: seasonal human coronavirus. HAdV: human adenovirus. HMPV: human metapneumovirus. HBoV: human bocavirus. †p values <0.01.

**
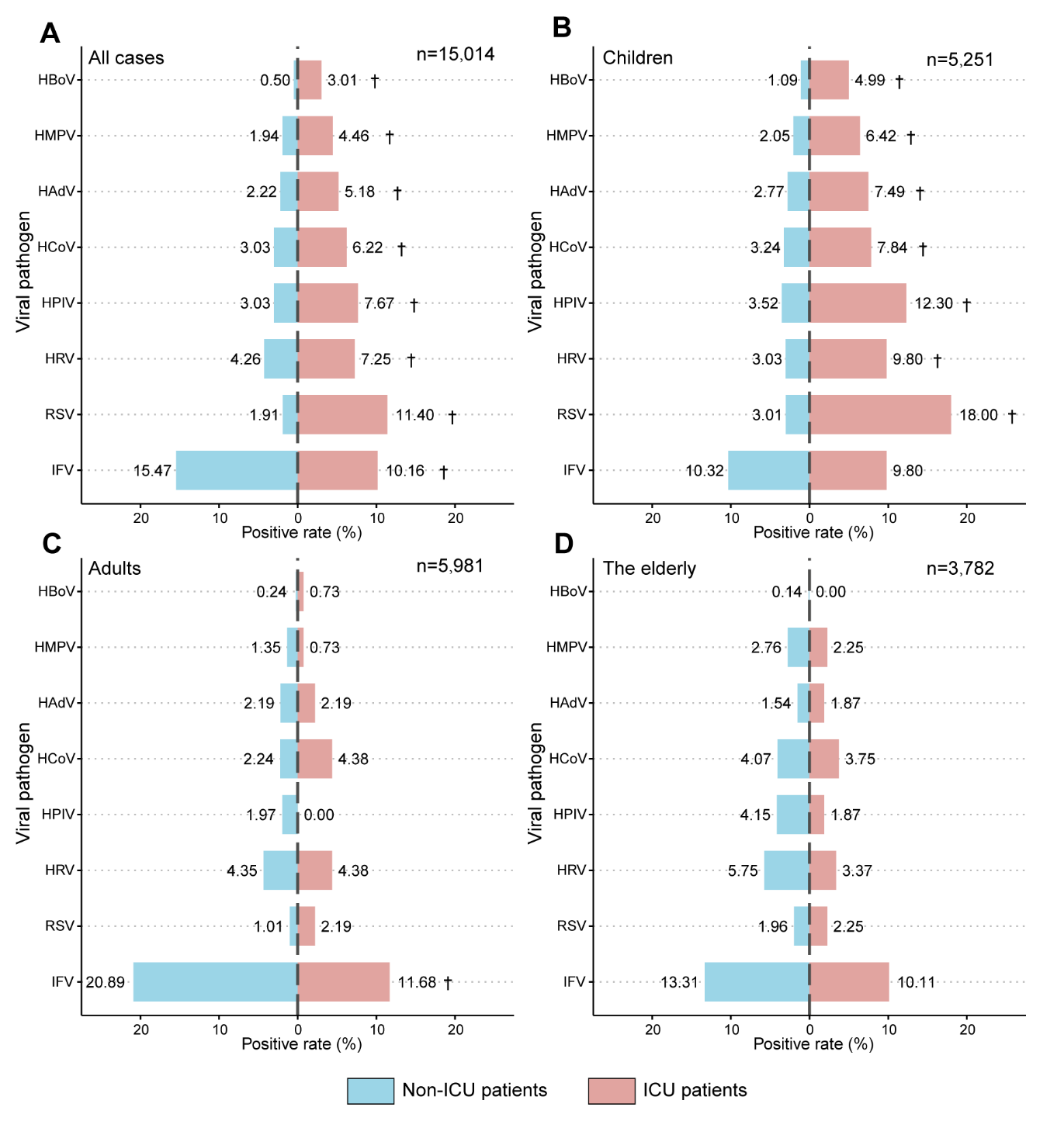
**

**Supplementary Figure 13. The co-infection pattern of ARIs patients by age group in Shanghai, eastern China.** (A) All cases. (B) Children. (C) Adults. (D) The elderly. The proportion of each detected pathogen in % is indicated by the length of colored bars. The red bar indicates viral co-infection and the blue bar indicates viral mono-infection.

**
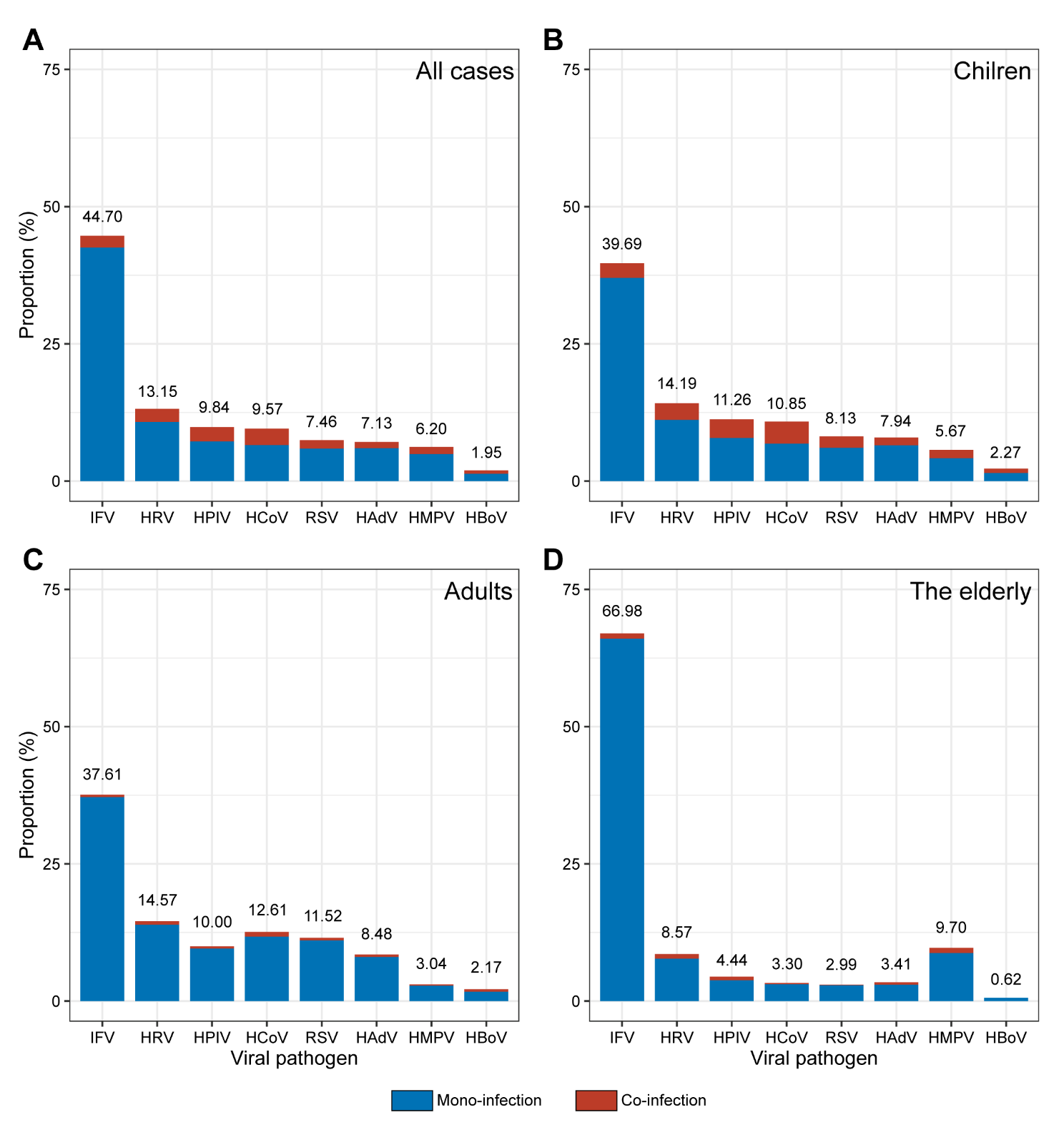
**
